# The first specific probe for pyrrolidine with multifunction by the interaction mechanism of atomic economic reaction

**DOI:** 10.1016/j.isci.2024.110024

**Published:** 2024-05-18

**Authors:** Xi-Ying Cao, Yan Huang, Si-Hong Chen, Shi-Wei Yu, Zu-Jia Chen, Zhong-Hao Li, Yu Zeng, Nan Chen, Liang Cao, Zhao-Yang Wang

**Affiliations:** 1 School of Chemistry, South China Normal University; Guangzhou Key Laboratory of Analytical Chemistry for Biomedicine; GDMPA Key Laboratory for Process Control and Quality Evaluation of Chiral Pharmaceuticals; Key Laboratory of Theoretical Chemistry of Environment, Ministry of Education, Guangzhou 510006, P.R. China; 2 The Education Ministry Key Lab of Resource Chemistry, Joint International Research Laboratory of Resource Chemistry of Ministry of Education, Shanghai Key Laboratory of Rare Earth Functional Materials, College of Chemistry and Materials Science, Shanghai Normal University, Shanghai 200234, P.R. China; 3 Shenzhen Key Laboratory of Cross-Coupling Reactions, Guangming Advanced Research Institute, Southern University of Science and Technology, Shenzhen 518055, P.R. China

**Keywords:** Natural sciences, Chemistry, Applied sciences

## Abstract

Pyrrolidine (PyD) has an important impact on the environment and human health. However, there is currently no method for trace detection of PyD. Here, we successfully designed diaminomethylene-4H-pyran (1) as the first specific fluorescent probe for PyD. Only by adding PyD to probe 1, there is blue fluorescence at 455 nm, and the color of the solution changes from colorless to yellow. The detection limit is 1.12 × 10^−6^ M, and the response time is less than 5 min. Meanwhile, probe 1 can also sense the gaseous PyD and detect PyD in actual water samples. Moreover, due to the low biological toxicity, probe 1 can detect the exogenous PyD in zebrafish. The preliminary mechanism shows that probe 1 and PyD undergo a combination-type chemical reaction to generate a new substance 1-PyD. Therefore, the 100% atom utilization reaction enables probe 1 to exhibit specific adsorption and removal of PyD.

## Highlights

• The first fluorescent probe sensing PyD in solution and gaseous states is reported
• This specific probe 1 with low LOD and fast response can detect PyD by naked eye
• Probe 1 with the multifunction of removing PyD can be used in actual water samples
• The low cytotoxicity makes probe 1 applied to detect exogenous PyD in zebrafish

## Graphical abstract

**Figure:**
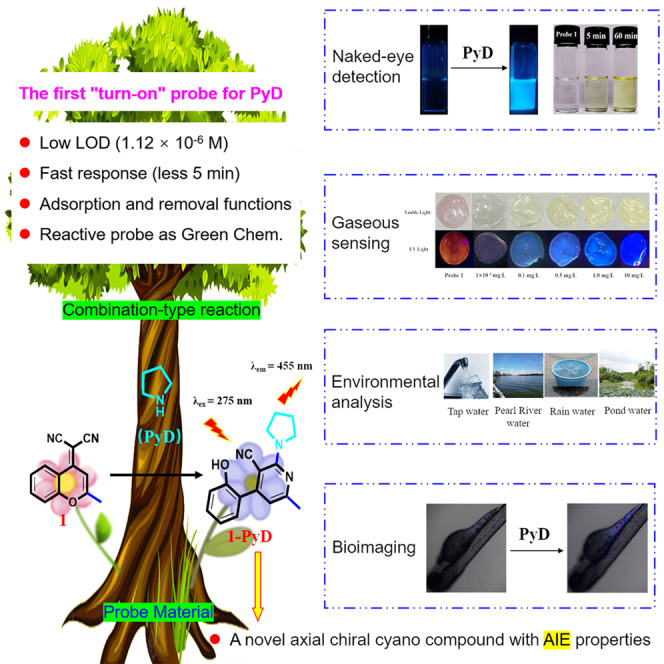

## Introduction

It is well-known that safety testing during the production, transportation, and use of bulk chemicals is a concern for everyone, not only including chemists. The structure unit of pyrrolidine (PyD) is widely found in many bio-active natural products^1^ and drug candidates.^2^ In fact, PyD is extensively used as an important synthon in organic synthesis.^3^^,^^4^^,^^5^^,^^6^ Not only PyD^7^ but its derivatives^8^ are also widely applied in the field of catalysis. However, we must know that, as an important fine chemical product with the annual consumption as high as 1.5 million tons worldwide, PyD is a toxic substance. Terribly, this fact has long been ignored by people. Perhaps it is related to the lack of effective testing methods. The semi-lethal concentration value (LC_50_) of PyD is 1.3 mg L^−1^, and its toxicity is higher than that of the common organic pesticides (e.g., trichlorfon) and insecticides. When the concentration of PyD is too high, it will affect the gastrointestinal mucosa, reduce the level of intracellular hemoglobin, and cause vascular disorders. Especially, PyD gas is corrosive to the skin mucous membranes and easy to cause conjunctivitis when irritating to the eyes. It has been reported that the recommended maximum allowable concentration of PyD in air is 0.1 mg m^−3^.^9^ Therefore, it is very necessary to design a probe for the targeted detection. And if there is an efficient and simultaneous removal of liquid and gaseous PyD while sensing, the users of this probe will be obviously happier and luckier. Unfortunately, although a few research groups have successfully reported the detection of PyD,^10^ the specificity of the developed probe for PyD is poor, and the sensing material is unable to remove PyD from the air, achieving the goal of purifying the air in time.

Reactive fluorescent probes are a class of probes designed on the basis of the specific reactivity of the analyte.^11^ The optical properties of the probe are changed by the chemical reaction between the analyte and the probe,^12^ so it has great advantages in specificity.^13^ Among the reactive fluorescent probes, usually, the probes with the interaction mechanism of the decomposition-type reaction are common,^14^ and have been used to specifically detect H_2_O_2_,^15^ H_2_S,^16^ and enzymes^17^ in living cells. In contrast, the reactive probes that are directly combined with the analyte to produce a substance are relatively rarely reported,^18^ and most of them are used to detect inorganic small molecule species, such as SO_2_,^19^ NO,^20^ H^+^,^21^ Hg^2+^,^22^ etc. Therefore, it is still challenging to develop probes for detecting organic small molecules by using the combination-type reaction with 100% atomic utilization.

Usually, the D-π-A type compounds containing the structure unit of dicynao-methylene-4H-pyran (DCM) have excellent optical properties, and their fluorescence can be regulated by adjusting the push-pull electronic structure.^23^^,^^24^^,^^25^ Therefore, many DCM compounds have been developed into the reactive fluorescent probes recently.^26^^,^^27^^,^^28^^,^^29^ It is a pity that most current reports on the sensing mechanism of probes with DCM unit are based on the decomposition-type reaction, thus hoping these probes to achieve the goal of complete adsorption and green removal of harmful substances is difficult and even theoretically impossible.

**Scheme 1:**
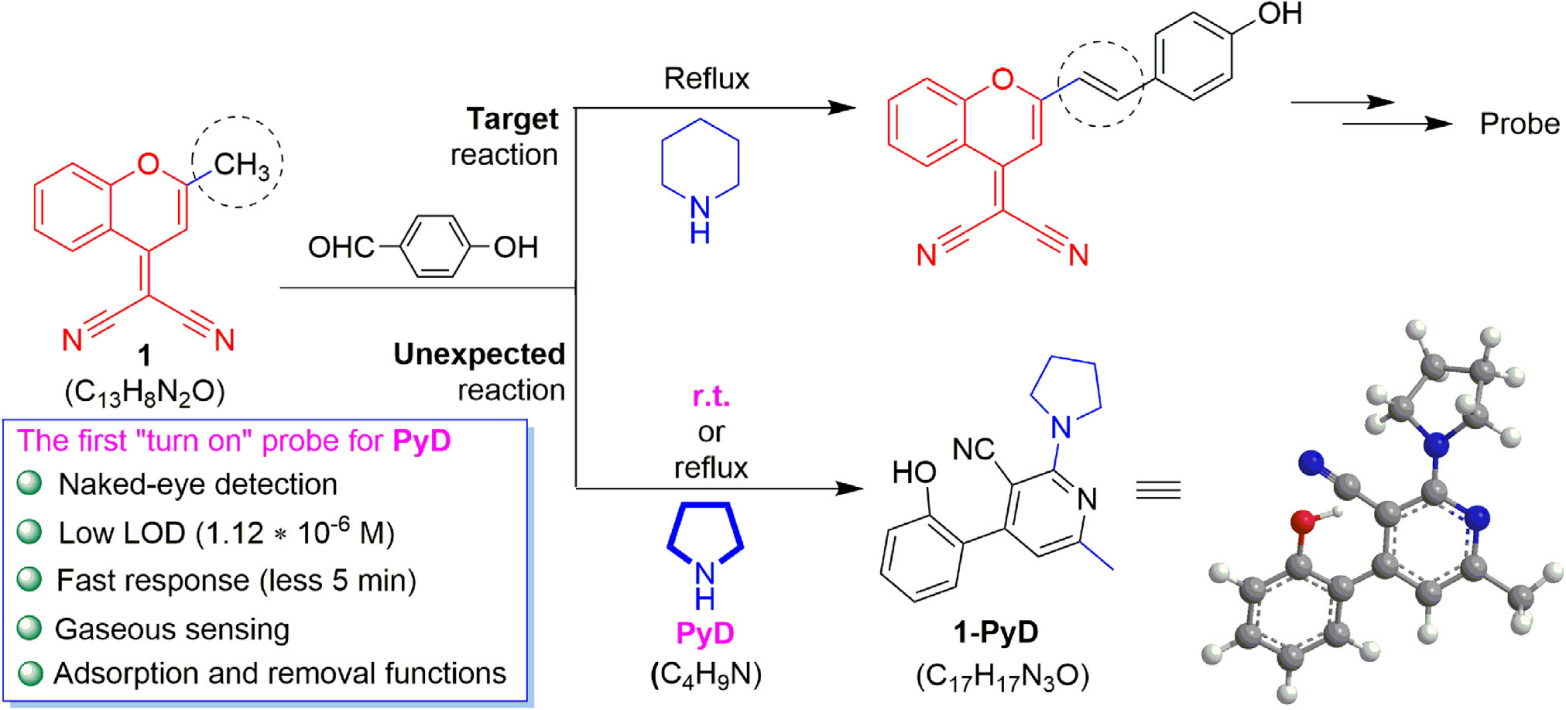
The reaction of probe 1 for PyD (For the specific synthesis program, single crystal analytical data and structural characterization of probe 1 and 1-PyD, see Schemes S1 and S2, Table S1; Figures S1 and S7–S13).

Being interested in the fluorescent detection of anions,^30^ cations,^31^ and organic small molecules,^32^ we used molecule 1 (which is a compound with DCM structure) as an intermediate to synthesize the related D-π-A type probe using organic base as a catalyst (Scheme 1). Unexpectedly, when replacing piperidine with PyD, once PyD is added into the acetonitrile solution of compound 1 at room temperature (RT), the color of the solution is quickly changed from colorless to yellow, and there is a blue fluorescence under a 365 nm UV lamp. Interestingly, a new compound 1-PyD can be obtained by purifying the reaction solution, and the single crystal test data show that 1-PyD is a product of combination-type reaction with 100% atomic utilization by the interaction of molecule 1 with PyD. Notably, molecule 1 and PyD can react in RT or heating environment, showing the same reaction phenomenon with the single crystal of the main product 1-PyD. Thus, we developed molecule 1 as the first combination-type reaction fluorescent probe with a “turn-on” response that can specifically detect and remove PyD (Scheme 1)

## Results and discussion

### Synthesis of probe 1 and preliminary theoretical calculation

The synthesis of probe 1 from compound III is shown as Scheme S1 in supplementary information. Both probe 1 and compound III are benzopyran derivatives, but they have different properties after introducing the dicynaomethylene structure. So, according to the density functional theory (DFT),^2^^,^^10^^,^^20^ we optimized their molecular configuration.

**Figure 1:**
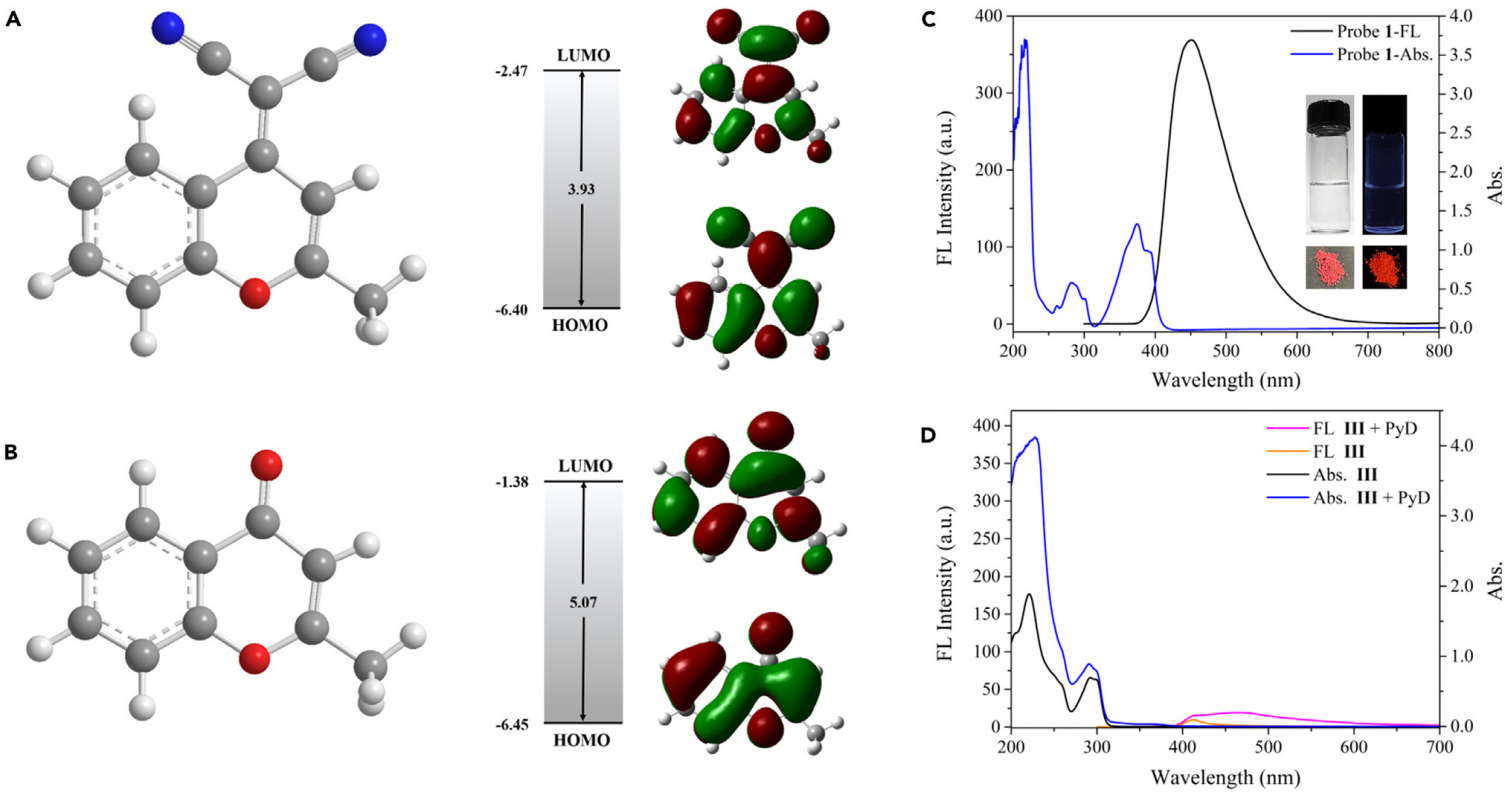
Comparison of band gap and basic optical properties between compound III and probe 1 (A–D) Molecular structure of probe 1 (A) and compound Ⅲ (B), and their optimized structure (B3LYP/6-31G); fluore-scence and UV spectra of probe 1 (Inset: Solution and solid images of probe 1 at 360 nm) (C); fluorescence and UV spectra of compound Ⅲ before and after interaction with PyD (D).

As shown in Figures 1A and 1B, the highest occupied molecular orbital/least unoccupied molecular orbital (HOMO/LUMO) energy band gaps (ΔE) calculated by Gaussian calculation are 3.93 and 5.07 eV, respectively. In general, the smaller the band gap, the longer the wavelength, and the higher the molecular reactivity.^33^^,^^34^^,^^35^ Here, this may be related to the introduction of the dicyano group into the molecular structure of probe 1, making the electron deficiency of probe 1 stronger. Therefore, it can be speculated that the activity of the reaction of probe 1 with PyD may be significantly higher than that of compound III.

In fact, the spectral data also show that there is no response between compound III and PyD at RT (Figure 1D). On the contrary, due to the π-π∗ transition within the molecule,^36^ the maximum UV absorption peak of probe 1 appears at 375 nm (Figure 1C). And under the excitation of λ_ex_ = 275 nm, the intramolecular aggregation-caused quenching (ACQ) effect results in the non-fluorescence in solution. However, there is a red fluorescence in the solid state.

### Sensing performance of probe 1

**Figure 2:**
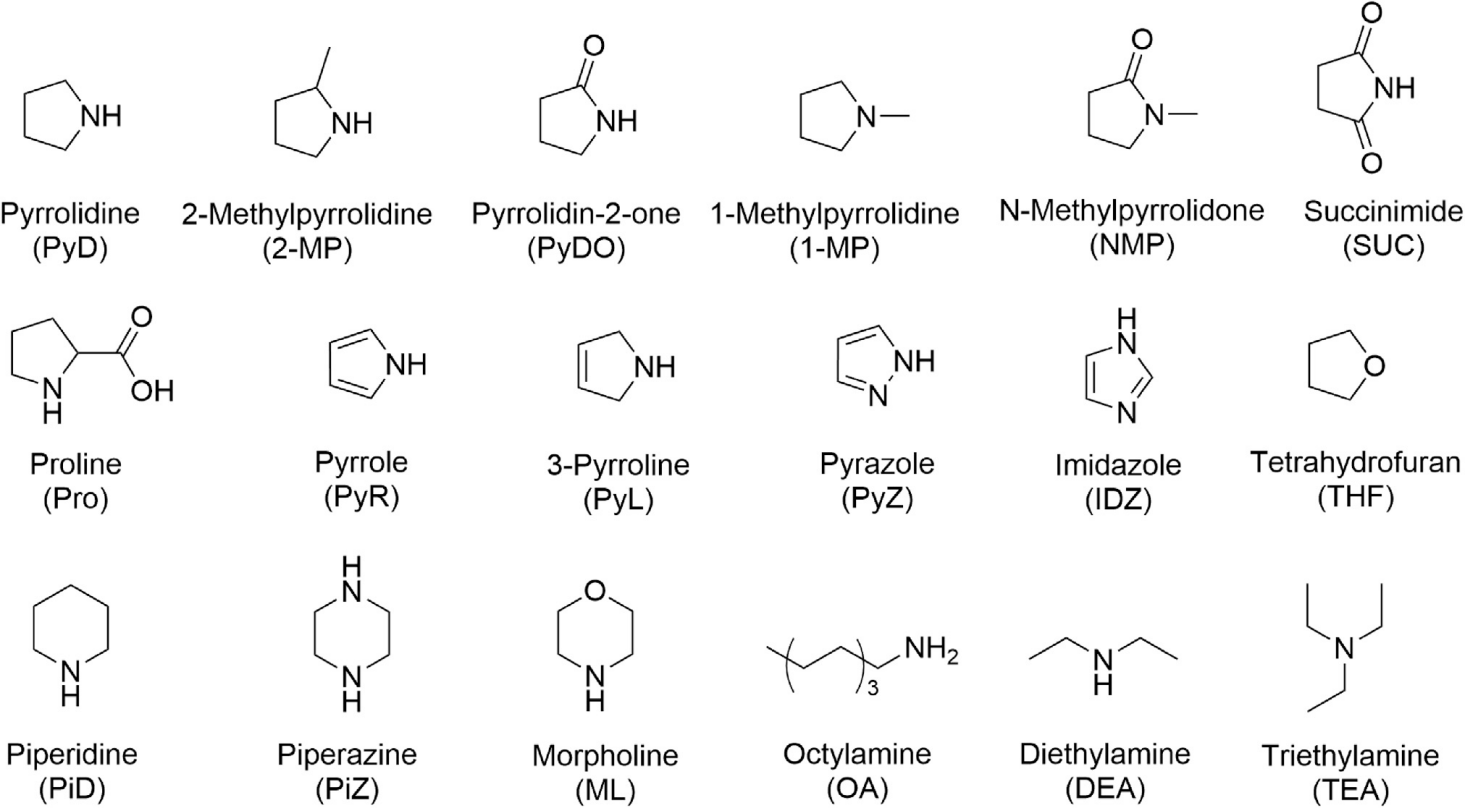
Structures of various amine analytes (including some typical compounds for contrast) and their abbreviations

**Figure 3:**
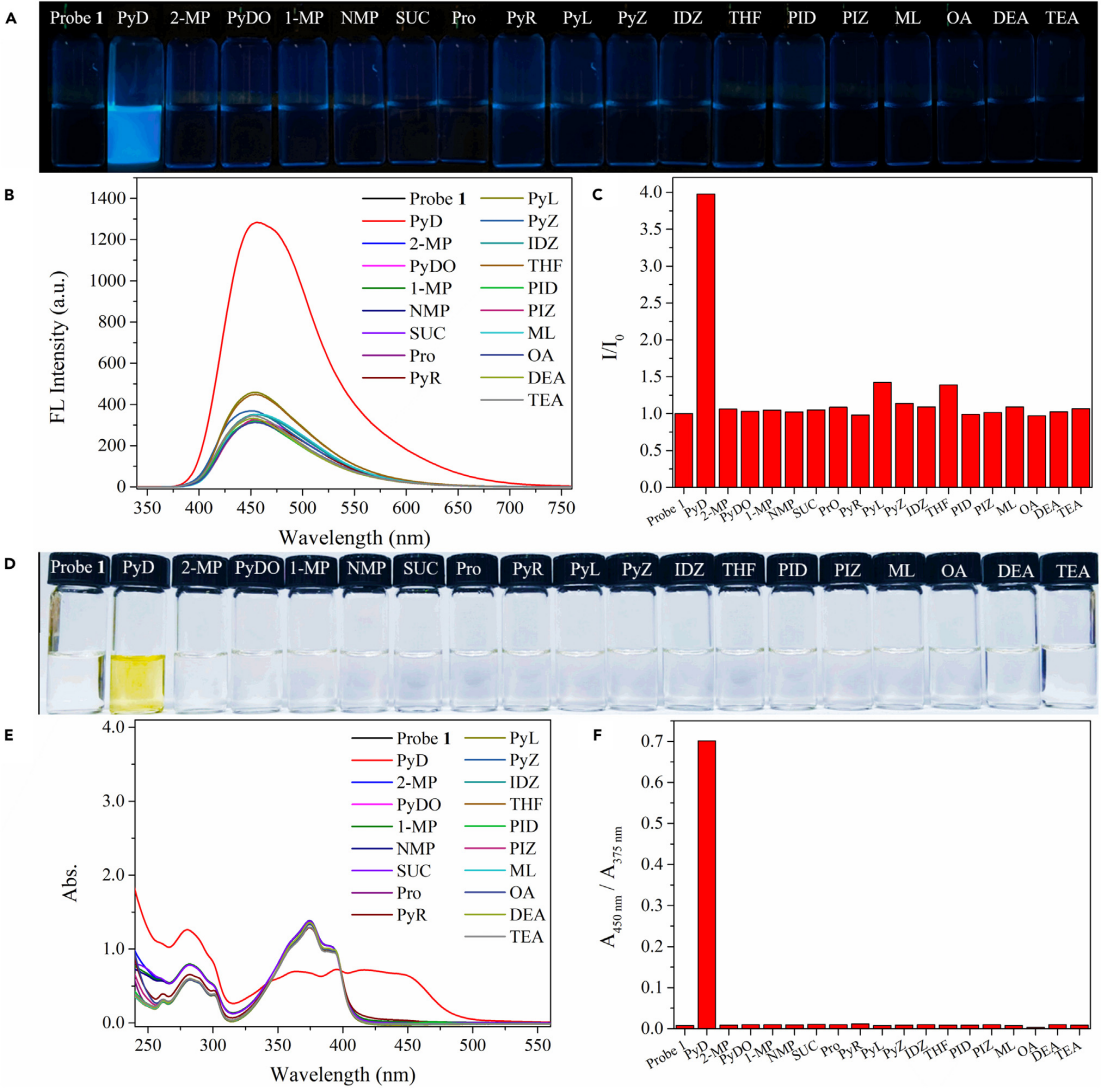
Fluorescence and UV selective detection of PYD by probe 1 (A–D) Fluorescence spectra (B and C) and UV spectra (E and F) of probe 1 (10^−4^ M in MeCN) after addition of different analytes, and color changes at UV 365 nm (A) and sunlight (D), respectively. (The possible interference experiments for probe 1 to detect PyD are shown in Figure S3).

The sensing performance of probe 1 on PyD was studied by using various cyclic amines and some typical compounds for contrast (their structures and the corresponding abbreviations are shown in Figure 2) as analytes. With acetonitrile as the solvent, the concentration of all analytes was configured as 0.2 M, then analytes of 10 equiv were added into the solution of probe 1 (10^−4^ M) respectively for fluorescence and UV analysis. The results are shown in Figure 3.

In acetonitrile solvent, probe 1 only displays a faint photoluminescence with an emission peak at 455 nm (λ_ex_ = 275 nm). Once adding PyD (10 equiv), the emission peak at 455 nm can be enhanced (Figure 3C). Notably, only the addition of PyD gives obvious blue fluorescence (Figures 3A and 3B). The UV absorption spectra also show that after the addition of PyD (10 equiv) for a while, the maximum absorption peak of probe 1 at 375 nm is gradually decreased, and there is a new absorption band around 450 nm (Figure 3E). Interestingly, the color of the solution is gradually changed from colorless to yellow (Figure 3D). These experimental results show that only PyD can interact with probe 1, resulting in the change of the optical properties.

Importantly, both the fluorescence emission spectra (Figure S3A) and UV absorption spectra (Figure S3B) of probe 1 solution (10^−4^ M in MeCN) in the presence of 10 equiv PyD and other organic analytes (10 equiv) show that there is no interference with the detection of PyD by probe 1 in the presence of other analytes. We speculate that this may be related to the density of the electron cloud on N and the steric hindrance of N in different amino compounds,^37^ resulting in the specific interaction mechanism of probe 1 with PyD. In addition, the interference of some common anions (Figure S3C) and metal ions (Figure S3D) also can be excluded. Therefore, probe 1 can be used as a specific “turn-on” fluorescent probe for the naked-eye detection of PyD.

### Titration experiments and limit of detection

**Figure 4:**
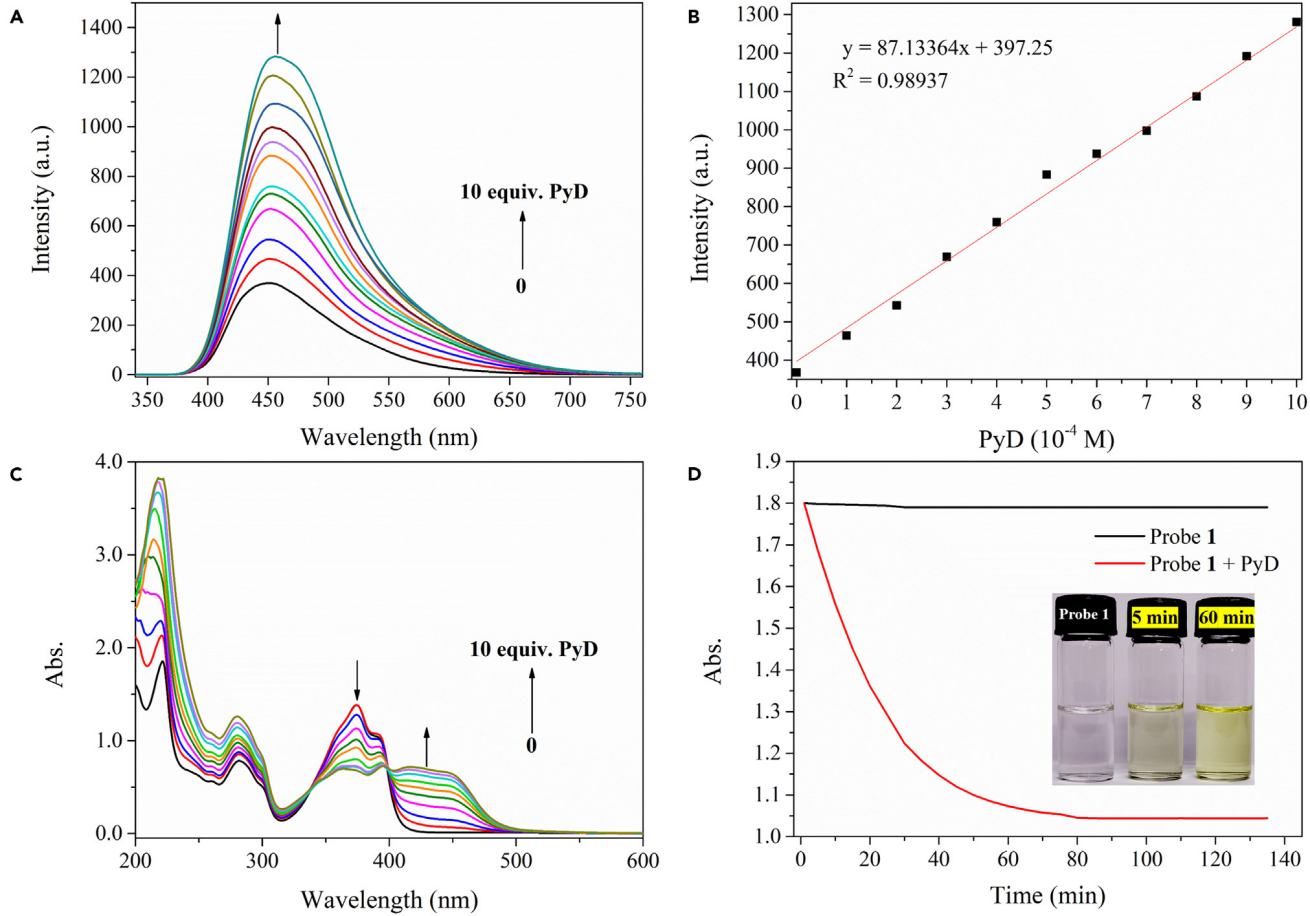
The change of the fluorescence spectra of the solution of probe 1 (10^−4^ M in MeCN) with the increase of PyD addition (0–10 equiv.) (A), the linear relationship between the concentration and the fluorescence intensity (455 nm) (B), and the change of UV absorption spectra (C) and response time (Inset: Color changes for 5 min and 60 min, respectively) (D). (The change of fluorescence intensity with time after the interaction between probe 1 and PyD is shown in Figure S2).

To further explore the efficiency of probe 1 in measuring PyD concentration, different concentrations of PyD (0–10 equiv) were successively added to the solution of probe 1 (10^−4^ M in MeCN). In order to ensure stability, each group of experiments was repeated three times, the reaction time was fixed at 1 h and the reaction temperature was RT. The results are shown in Figure 4.

With the increase of PyD concentration, the fluorescence intensity of probe 1 at 455 nm is increased with the emission of blue fluorescence (Figure 4A). And there is a good linear relationship between the fluorescence intensity at 455 nm and the concentration of PyD (R^2^ = 0.98937), as shown in Figure 4B. With these data in hand, the limit of detection (LOD) is calculated to be 1.12 × 10^−6^ M (based on LOD = 3*δ*/K^38^^,^^39^), indicating that the sensitivity is high.

The UV absorption spectra of probe 1 solution show a ratiometric change with the increase of PyD concentration, as shown in Figure 4C, and an isoabsorption point can be formed at 400 nm. This fully indicates that probe 1 may interact with PyD to produce a new substance. At the same time, the color of the reaction system is changed from colorless to yellow Figure 4D). The fluorescence intensity also gradually increased over time, as shown in Figure S2. Therefore, for the first time, this study can realize the specific naked-eye detection of low concentration PyD.

Response time is another important parameter of the probe real-time tracking target.^1^ Therefore, we also evaluated the time dependence of the UV intensity change of probe 1 in the presence of PyD. The results show that the color of the solution of probe 1 changed significantly after adding PyD for 5 min and basically reached stability after 60 min (Figure 4D). Compared with the probes for the detection of *N*-containing analytes reported in recent years,^40^^,^^41^^,^^42^^,^^43^^,^^44^ our probe 1 has certain advantages in terms of sensitivity, selectivity, and response time (Table S2).

### Response mechanism of probe 1 for PyD

As mentioned before in Scheme 1, the new compound 1-PyD can be isolated from the reaction as the product of probe 1 and PyD (Scheme S2). Its structure is systematically analyzed by ^1^H NMR, ^13^C NMR, and electrospray ionization mass spectrometry (ESI-MS). Their data can be seen in supplemental information, and the spectra are shown in Figures S9–S11), especially X-ray single crystal analysis (Table S1; Figure S1).

At the same time, ESI-HRMS was used to qualitatively monitor the reaction products between probe 1 and PyD. In order to obtain faster and better results, PyD (10 equiv) was added to probe 1 (10^−4^ M, MeCN) solution at RT for reaction for 1 h. The result is shown in Figure S12. Obviously, a new peak can be detected at 280.1443 (*m/z*) as [M + H]^+^ indeed, which is in good agreement with the theoretically calculated peak of the ring-opening product 1-PyD, 280.1444 (*m/z*) as [M + H]^+^.

**Scheme 2:**
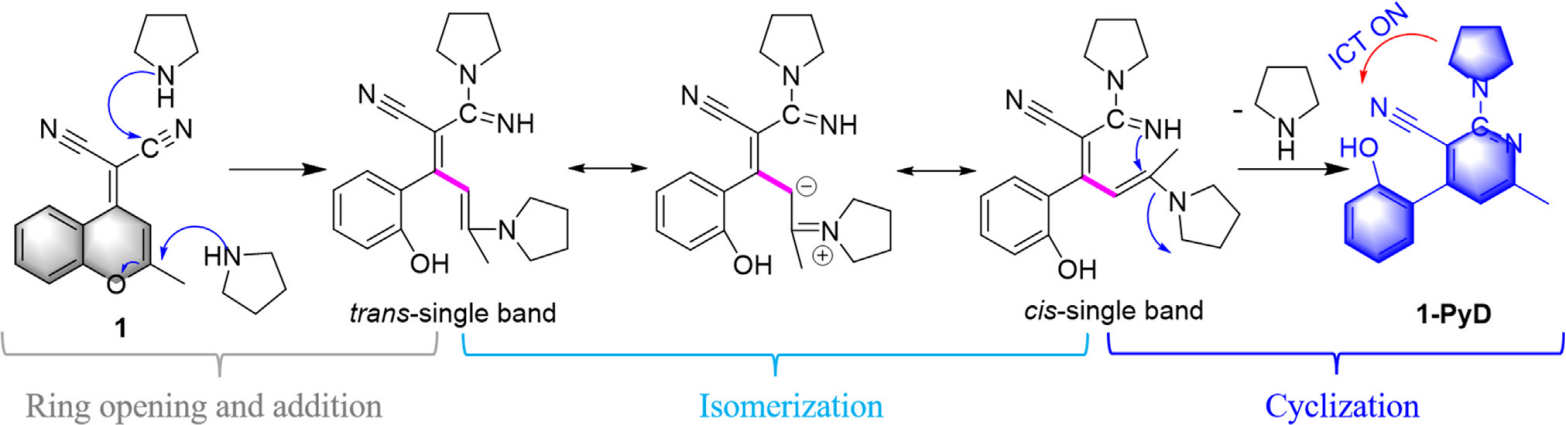
The possible mechanism of interaction between probe 1 and PyD (For the verification of probe 1 and PyD reaction mechanism, see Scheme S3 and Figures S14–S19).

Thus, based on the chemical reaction of probe 1 with PyD to form 1-PyD, we proposed a possible interaction mechanism for this detection (Scheme 2). Therein, the original ring structure of probe 1 is opened and a new *N*-containing aromatic ring is generated. Referring to the reaction mechanism in the literature reported before,^45^^,^^46^^,^^47^^,^^48^ in this sensing process, the detection mechanism should be involved in three steps as the order of ring opening, isomerization, and cyclization accompanied by aromatization, and the latter may be one of the driving forces of reaction.

What’s more; the aforementioned possible transformation mechanism is also confirmed by some validation experiments. Firstly, when probe 1 and PyD are refluxed in MeCN for 30 min (Scheme S2), the new compound 1-PyD shown in Scheme 2 can be obtained with a yield of 51%. And during the reaction, the color of solution is significantly changed from red to yellowish, and the fluorescence color is changed from colorless to blue. These results are in good agreement with the aforementioned and prove the detection mechanism.

Delightedly, similar results can be obtained when compound 1Me (Scheme S3) is used as an analog of probe 1 in this transformation (the data and spectra of structure characterization for the corresponding product 1Me-PyD can be seen in supplementary information).

On the other hand, the crystal structure (Figure S4) shows that the 1-PyD molecule is a non-planar molecule. The dihedral angle between the benzene ring and the pyridine ring in the molecule is 53.780°. The molecule can rotate freely within the molecule, and the molecules in the aggregated state are randomly sorted. There is no π-π stacking between molecules, which makes compound 1-PyD have certain aggregation-induced emission (AIE) properties^49^^,^^50^ (the results of AIE characterization experiment can be seen in Figure S5).

In addition, the calculation of the HOMO and LUMO orbital electron distribution of 1-PyD molecules by DFT (Figure S4C) shows that the electrons in HOMO orbitals are mainly distributed on the pyrrolidine and pyridine groups, while the electrons in LUMO orbitals are mainly distributed on the benzene ring and pyridine groups, and the spatial separation of the HOMO and LUMO is beneficial for intramolecular charge transition (ICT).^51^ Thus, due to this significant ICT effect, the product 1-PyD has blue fluoresce at 455 nm under λ_ex_ = 275 nm (Scheme 2).

Shortly, from different perspectives, we investigated the interaction mechanism of probe 1 with PyD. Importantly, these new interesting studies also show that the tandem reaction in Scheme 2 may be applied to some new axially chiral cyano compounds with AIE properties,^52^^,^^53^^,^^54^ which may bring new synthesis strategies for potential functional molecules. Now, our laboratory is conducting more in-depth work.

### Detection application of gaseous PyD

Because of the volatility and toxicity of PyD, there is a need to develop a portable method for PyD detection in the gaseous state. A simple organic detection film was prepared by using polyvinyl alcohol (PVA) to load probe 1 as the reported method.^15^

**Figure 5:**
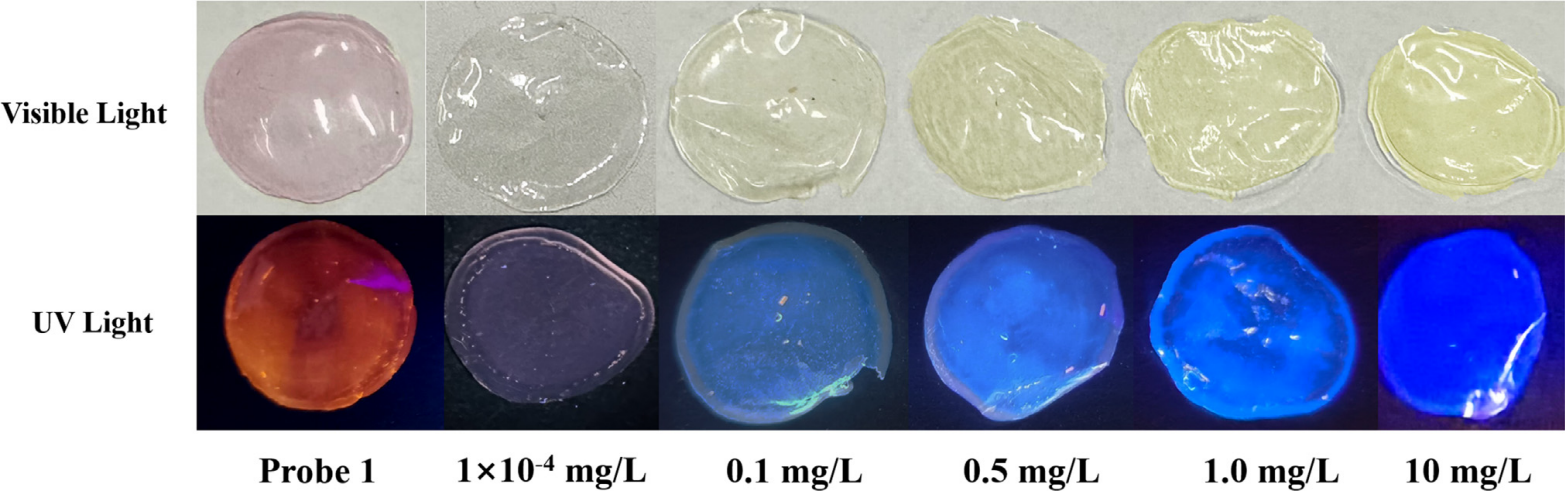
The color change of film material based on probe 1 at different concentrations of gaseous PyD under visible light or UV lamp

As shown in Figure 5, the detection film is pink in visible light and has red fluorescence under a 365 nm UV lamp. Once the detection film is exposed to different concentrations of gaseous PyD (0.1–10 mg L^−1^),^43^ the color of the detection film is gradually changed from pink to yellow under visible light, and its fluorescence color is gradually changed from red to dark blue.

Notably, even at a low PyD concentration of 0.1 mg L^−1^, the significant change also can be observed. This means that the concentration observed by the naked eye is very close to the life-threatening concentration of PyD gas mentioned in the literature.^9^

Therefore, the portable detection film has a high sensitivity to the detection of gaseous PyD, and there is a great application value for detecting PyD that evaporates or leaks during the use involving in PyD. And its adsorption effect derived from the response mechanism is of great significance for the protection of human health.

### Detection of PyD in actual water samples

PyD is water soluble, so it also has a certain pollution to water quality in some cases. In order to verify the feasibility in detecting PyD in actual water samples with probe 1, PyD in tap water, Pearl River water, rain water, and pond water were labeled and analyzed as the reported method.^55^^,^^56^ The labeled sample was fully responded at RT and the output data of ultraviolet absorption spectrum were recorded. All data were replicated with a relative standard deviation (RSD) within 0.03–1.15%, indicating good reproducibility of the data. And all of the actual samples had recoveries within 86.00–105.24%. The aforementioned results reveal that probes 1 show satisfactory reliability and feasibility for the analysis of PyD in real samples (Table 1).

**Table 1:** Results of the detection of PyD in water samples by probe 1

Sample	The added (10^−4^ M)	The found (10^−4^ M)	Recovery (%)	RSD (%) (*n* = 3)
Tap water	2.50	2.45, 2.47, 2.46	98.08, 98.91, 98.34	0.36
	5.00	4.45, 4.51, 4.41	89.02, 90.16, 88.27	0.87
	10.00	9.63, 9.66, 9.76	96.26, 96.57, 97.57	0.57
Pearl River water	2.50	2.61, 2.62, 2.62	104.56, 104.77, 104.77	0.08
	5.00	4.53, 4.49, 4.47	90.70, 89.85, 89.36	0.61
	10.00	9.89, 9.72, 9.67	98.86, 97.20, 96.67	0.96
Rain water	2.50	2.23, 2.17, 2.23	89.16, 86.93, 89.16	1.15
	5.00	4.34, 4.30, 4.30	86.85, 86.00, 86.00	0.46
	10.00	10.08, 9.99, 9.99	100.77, 99.93, 99.93	0.26
Pond water	2.50	2.63, 2.61, 2.63	105.24, 104.42, 105.24	0.35
	5.00	4.39, 4.37, 4.36	87.72, 87.41, 87.19	0.25
	10.00	10.08, 10.09, 10.08	100.83, 100.86, 100.83	0.03

### Fluorescent imaging in zebrafish

To achieve the detection of toxic PyD in fish in contaminated water, we investigated its ability to image PyD in living organisms *in vivo*, building on the results of our previous experiments. We selected zebrafish, a commonly used fluorescent imaging model organism that shares many genes with humans,^57^^,^^58^^,^^59^ as the subject of our research.

Firstly, the toxicity of probe 1 to zebrafish was assessed. As the results shown in Figure S6, probe 1 has negligible toxicity to zebrafish in a large concentration range (0–300 μM). Thus, probe 1 can be readily applied in zebrafish bioimaging.

**Figure 6:**
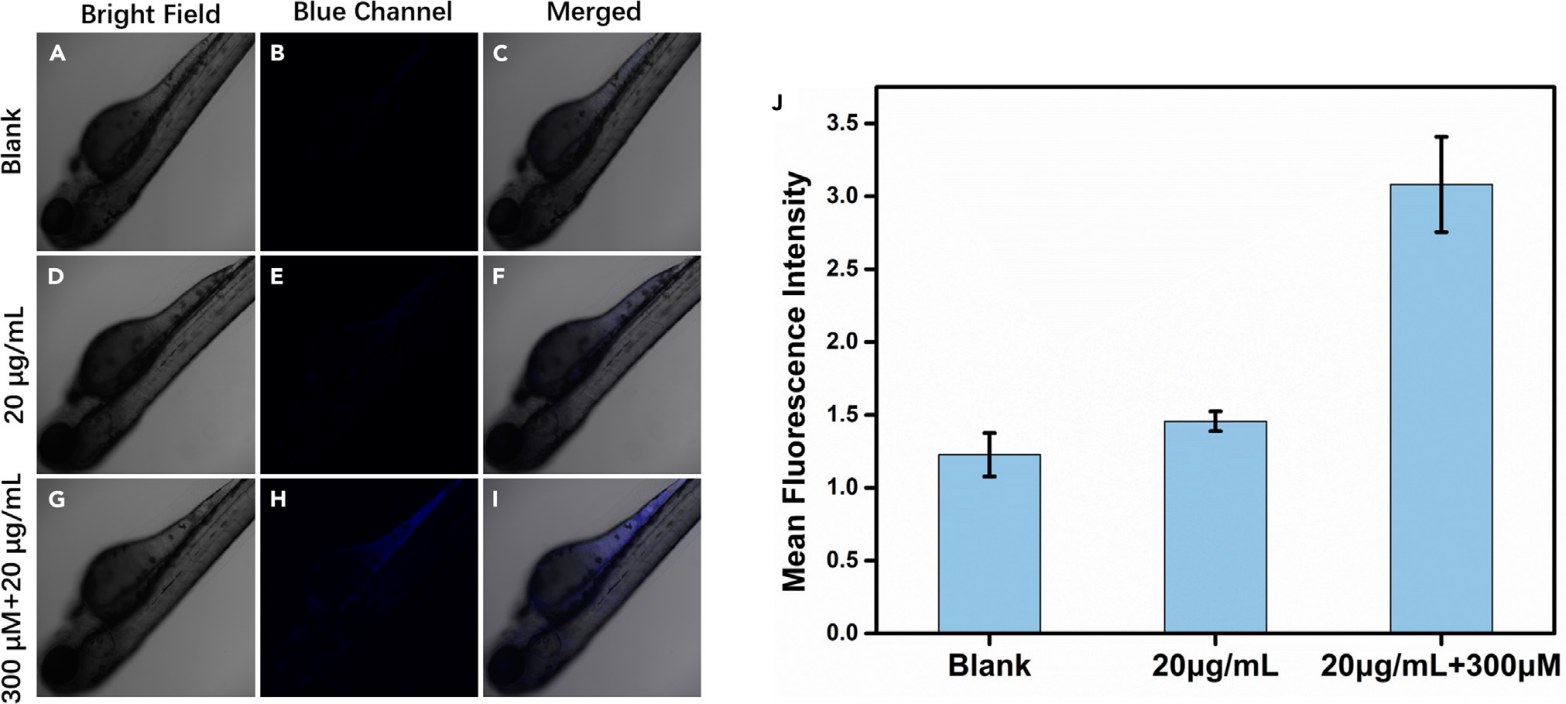
Fluorescence imaging of zebrafish (A–J) Fluorescence image of zebrafish (A–C); Control group: the live intact zebrafish embryos was fed with probe 1 (20.0 μg mL^−1^) and cultured for 1 h (D–F); Experiment group: the zebrafish embryos were fed with PyD (300 μM) and cultured for 1 h, and then fed with probe 1 (20.0 μg mL^−1^) and cultured for 1 h (G–I); And fluorescence imaging histogram (J). (Blue channel: λ_ex_ = 405 nm, λ_em_ = 410–485 nm). Scale bar: 250 μm. (Survival rates of zebrafishes treated with different concentrations of probe 1 are shown in Figure S6).

Secondly, as shown in Figure 6, it can be observed in a special cell culture medium that after incubating the probe 1 (20.0 μg mL^−1^) with zebrafish lacking melanin for 1 h, just faint blue fluorescence can be detected. However, after pre-incubating the probe 1 (20.0 μg mL^−1^) with zebrafish for 1 h and then adding PyD (300.0 μM) and incubating for 1 h, there is significant blue fluorescence in the abdomen and cranium of zebrafish.

These findings demonstrate that probe 1 can perform fluorescence imaging of PyD *in vivo* in zebrafish, suggesting that it has the potential to image PyD in intricate biological systems.

### Conclusions

In summary, based on an unexpected reaction, we found that the “old-molecule” compound 1 with DCM unit has a specific response to PyD. Thus, for the first time, we developed a green combination-type fluorescent probe 1 that can detect and adsorb PyD simultaneously. As the first selective probe for PyD, probe 1 is a “turn-on” reactive fluorescent probe, it can be used for the detection of PyD both in solution and gaseous state. It is worth noting that, the product 1-PyD after the reaction of probe 1 with PyD is very atomic economy. Therefore, for PyD existing in the environment, probe 1 has the dual significance of color monitoring and adsorption removal making it more in line with the concept of green chemistry. At the same time, probe 1 has low cytotoxicity and can be used to detect exogenous PyD in zebrafish. In short, this study is beneficial in promoting the development of economical reaction-type probes and enriching the content of green chemistry in multiple aspects and levels.

### Limitations of the study

Our research has certain limitations to a certain extent. Probe 1 and other organic amines, such as pyrroline, also have a certain response for a long time. And the reaction mechanism between pyrrolidine and probe 1 needs to be further explored.

## STAR★Methods

### Key resources table

**Table:** 

REAGENT or RESOURCE	SOURCE	IDENTIFIER
**Chemicals, peptides, and recombinant proteins**		

Ethyl acetate	Guangzhou chemical reagent factory	CAS: 141-78-6
dichloromethane	Annaiji (Shanghai) Pharmaceutical Chemical Co., Ltd	CAS: 75-09-2
petroleum ether	Annaiji (Shanghai) Pharmaceutical Chemical Co., Ltd	CAS: 8032-32-4
malononitrile	Annaiji (Shanghai) Pharmaceutical Chemical Co., Ltd	CAS: 109-77-3
pyrrolidine (PyD)	Annaiji (Shanghai) Pharmaceutical Chemical Co., Ltd	CAS: 123-75-1
pyrrole (PyR)	Annaiji (Shanghai) Pharmaceutical Chemical Co., Ltd	CAS: 109-97-7
1-methylpyrrolidine (1-MP)	Annaiji (Shanghai) Pharmaceutical Chemical Co., Ltd	CAS: 120-94-5
2-methylpyrrolidine (2-MP)	Annaiji (Shanghai) Pharmaceutical Chemical Co., Ltd	CAS: 765-38-8
pyrrolidin-2-one (PyDO)	Annaiji (Shanghai) Pharmaceutical Chemical Co., Ltd	CAS: 616-45-5
succinimide (SUC)	Annaiji (Shanghai) Pharmaceutical Chemical Co., Ltd	CAS: 123-56-8
pyrazole (PyZ)	Annaiji (Shanghai) Pharmaceutical Chemical Co., Ltd	CAS: 288-13-1
diethylamine (DEA)	Annaiji (Shanghai) Pharmaceutical Chemical Co., Ltd	CAS: 107-15-3
imidazole (IDZ)	Annaiji (Shanghai) Pharmaceutical Chemical Co., Ltd	CAS: 288-32-4
proline (Pro)	Annaiji (Shanghai) Pharmaceutical Chemical Co., Ltd	CAS: 609-36-9
triethylamine (TEA)	Annaiji (Shanghai) Pharmaceutical Chemical Co., Ltd	CAS: 121-44-8
2-Hydroxy acetophenone	Bide Pharmatech Ltd.	CAS: 582-24-1
Acetonitrile	Guangzhou Chemical Reagent Factory	CAS: 75-05-8
acetic anhydride	Guangzhou Chemical Reagent Factory	CAS: 108-24-7
tetrahydrofuran (THF)	Guangzhou Chemical Reagent Factory	CAS: 109-99-9
Octylamine (OA)	Guangzhou Chemical Reagent Factory	CAS: 111-86-4
Concentrated hydrochloric acid	Guangzhou Chemical Reagent Factory	N/A
Methanol	Tianjin Zhiyuan Chemical Reagent Co., Ltd.	CAS: 67-56-1
sodium sulfate	Tianjin Zhiyuan Chemical Reagent Co., Ltd.	CAS: 15124-09-1
sodium bicarbonate	Tianjin Zhiyuan Chemical Reagent Co., Ltd.	CAS: 144-55-8
sodium hydroxide	Tianjin Zhiyuan Chemical Reagent Co., Ltd.	CAS: 1310-73-2
3-Pyrroline (PyL)	Beijing InnoChem Science & Technology Co., Ltd.	CAS: 109-96-6
piperidine (PiD)	Beijing InnoChem Science & Technology Co., Ltd.	CAS: 110-91-8
piperazine (PiZ)	Beijing InnoChem Science & Technology Co., Ltd.	CAS: 110-89-4
morpholine (ML)	Beijing InnoChem Science & Technology Co., Ltd.	CAS: 110-85-0
Zebrafish	Shandong Yixiyue Biological Technology Co., Ltd.	N/A

**Deposited data**		

Raw and analyzed data	This paper	N/A

### Resource availability

#### Lead contact

Further requests for resources regarding this study will be fulfilled by the corresponding author, Zhao-Yang Wang (wangzy@scnu.edu.cn).

#### Materials availability

This work did not produce any new unique reagents.

#### Data and code availability

• The data reported in this paper will be shared by the lead contact upon request.
• This paper does not report original code.
• Any additional information required to reanalyze the data reported in this paper is available from the lead contact upon request.

### Experimental model and study participant details

There are no experimental model and study participant details to be reported.

### Method details

#### Materials

Ethyl acetate, dichloromethane, petroleum ether, malononitrile, pyrrolidine (PyD), pyrrole (PyR), 1-methylpyrrolidine (1-MP), 2-methylpyrrolidine (2-MP), pyrrolidin-2-one (PyDO), succinimide (SUC), pyrazole (PyZ), diethylamine (DEA), imidazole (IDZ), proline (Pro), triethylamine (TEA) were purchased from Annaiji (Shanghai) Pharmaceutical Chemical Co., Ltd.; 2-Hydroxy acetophenone was purchased from Bide Pharmatech Ltd.; Acetonitrile, acetic anhydride, tetrahydrofuran (THF), Octylamine (OA), concentrated hydrochloric acid were purchased from Guangzhou Chemical Reagent Factory; Methanol, sodium sulfate, sodium bicarbonate, sodium hydroxide were purchased from Tianjin Zhiyuan Chemical Reagent Co., Ltd.; 3-Pyrroline (PyL), piperidine (PiD), piperazine (PiZ), morpholine (ML) were purchased from Beijing InnoChem Science & Technology Co., Ltd.; Zebrafish were purchased from Shandong Yixiyue Biological Technology Co., Ltd.

#### Conventional reagents and equipment

Flash column chromatography was performed using 200–300 mesh silica gel. The ^1^H and ^13^C NMR spectra were acquired on a 600 MHz Bruker spectrometer. High resolution mass spectra (HR-MS) were recorded on the MAT95XP high resolution mass spectrometry (Thermo Fisher Technologies, USA). UV-Visible absorbance spectra were performed on UV-2700 ultraviolet spectrometer (SHIMADZU, Japan). The fluorescence spectra were measured by F-4600 fluorescence spectrometer (HITACHI, Japan). The biological imaging was monitored with a laser confocal microscope (Keyens Co., LTD., China). The single crystal structure was determined by Agilent Gemini E type X-ray single crystal diffraction (Agilent, USA) using Mo as a target.

#### General procedure for optical spectral measurements

The samples of probe **1** were dissolved in MeCN to acquire stock solution. Then, the test solution of probe **1** (10^−4^ M) was prepared for fluorescence emission spectra at room temperature.^60^

#### Limit of detection

As the reported method,^61^ the limit of detection (LOD) was measured by the following equation:

LOD=3δ/K.

Therein, *δ* is the standard deviation of the blank measurements (*n* = 10), and the *K* is the slope of the calibration curve.

#### Preparation of polyvinyl alcohol membrane material

Referring to the reported method,^15^ 8 mg of polyvinyl alcohol (PVA) and 2 mg of probe **1** were added into 90 mg of water. The mixture was thoroughly stirred at room temperature until complete dissolution. Then, the obtained solution was dropped into a specific mold. After drying in the oven, the PVA film material loaded with probe **1** can be prepared.

#### Zebrafish culture and imaging

In order to detect the exogenous PyD in zebrafish embryos, the fluorescence imaging experiments were carried out with reference to the method reported before.^19^^,^^21^

The embryos were incubated in a culture medium at 28°C for 24 h and then transferred to a 24-well plate. The solution of probe **1** (20 *μ*g mL^−1^) was added and incubated for 1 h, and then rinsed with the fresh medium for 3 times.

After the treatment of being incubated with PyD (300 *μ*M) for 1 h, and rinsed with fresh medium three times, the embryos were placed under a laser confocal microscope for imaging.

All zebrafish were purchased from ShanghaiJieSiJie Laboratory Animal Co.,Ltd., and all the animal experiments strictly abide by animal ethics regulations of Shanghai Normal University.

#### Synthesis of probe 1

According to the synthesis method in the literature,^62^ probe **1** was synthesized using compound **I** as starting material as Scheme S1. The structure of all synthesized compounds was well characterized, and their characterization data were in agreement with those of the known compounds in the literature. Taking compound **III** and probe **1** as examples.

#### 2-Methyl-4H-chromen-4-one (compound III)

After compound **II** (14 mmol, 2.4946 g) was completely dissolved in methanol (30 mL), the concentrated HCl (12 M, 1 mL) was slowly added. The mixture was stirred at room temperature for 14 h. When the reaction was completed, the solvent was removed by a rotary evaporator. The residue was dissolved in 50 mL of ethyl acetate and wash with the saturated sodium bicarbonate solution, the deionized water and the saturated sodium chloride solution, respectively. The organic phase was dried with anhydrous Na_2_SO_4_. After removing the solvent *in vacuo*, the crude product was purified via silica gel column chromatography, by eluting with petroleum ether and Et_2_O to afford compound **III** as a colorless solid.

Characterization data: 2.1079 g, yield 94%; m.p.: 70.2°C–70.8°C (Reference data^63^: 71°C–72°C); ^**1**^**H NMR** (600 MHz, CDCl_3_), *δ*, ppm: 2.39 (*s*, 3H), 6.19 (*s*, 1H), 7.36–7.40 (*m*, 1H), 7.42 (*d*, *J* = 8.4 Hz, 1H), 7.62–7.65 (*m*, 1H), 8.18 (*d*, *J* = 6.0 Hz, 1H); **ESI-HRMS**, *m*/*z*: Calcd for C_10_H_9_O_2_ [M + H]^+^, 161.0595, found: 161.0597.

#### 2-(2-Methyl-4H-chromen-4-ylidene)malononitrile (probe 1)

Malononitrile (0.3633 g, 5.5 mmol) was added into the solution of compound **III** (0.7592 g, 4.74 mmol) in Ac_2_O (10 mL). The mixed solution was refluxed for 14 h. When the reaction was completed, the reaction mixture was concentrated *in vacuo*. Then, H_2_O (20 mL) was added into the residue. After stirring at 100°C for 30 min, the obtained mixture was extracted with DCM (3 × 30 mL). The combined organic layer was washed with the saturated Na_2_CO_3_ solution (100 mL), and dried over Na_2_SO_4_. After removing the solvent *in vacuo*, the crude product was purified via silica gel column chromatography, by eluting with petroleum ether and DCM to afford probe **1** as pale red crystal.

Characterization data: 0.8587 g, yield 87%; m.p.: 182.5°C–183.2°C (Reference data^24^: 184°C–186°C): ^**1**^**H NMR** (600 MHz, CDCl_3_), *δ*, ppm: 2.46 (s, 3H), 6.73 (s, 1H), 7.45–7.49 (m, 2H), 7.74 (m, 1H), 8.92 (*d*, *J* = 8.4 Hz, 1H); **ESI-HRMS**, *m*/*z*: Calcd for C_13_H_8_N_2_O [M + H]^+^, 209.0709, found: 209.0707.

#### Synthesis of 1-PyD

The compound **1-PyD** was unexpectedly obtained in the reaction shown as Scheme S2. The probe **1** (0.1040 g, 0.5 mmol) was dissolved in acetonitrile (10 mL), and then pyrrolidine (41 *μ*L, 0.5mmol) was added. After the reaction at room temperature for 2 h (or refluxing for 30 min, when used as a validation experiment), the reaction solution was concentrated under reduced pressure. And the crude product was purified by silica gel column chromatography to give **1-PyD** as a colorless solid，.

Characterization data: m.p. 169.0°C–170.4°C; ^**1**^**H NMR** (600 MHz, CDCl_3_), *δ*, ppm: 1.91 (*t*, *J* = 6.6 Hz, 4H), 2.47 (*s*, 3H), 3.55 (*t*, *J* = 6.6 Hz, 4H), 6.95 (s, 1H) 7.18–7.24 (*m*, 2H), 7.40–7.45 (*m*, 1H), 7.85 (*d*, *J* = 7.8 Hz, 1H); ^**13**^**C NMR** (150 MHz, CDCl_3_), *δ*, ppm: 25.2, 25.5, 50.5, 96.2, 103.4, 117.1, 117.2, 123.7, 123.9, 131.3, 144.7, 152.6, 158.1, 160.0; **ESI-HRMS**, *m*/*z*: Calcd for C_13_H_8_N_2_O [M + H]^+^, 208.1444, found: 208.1441.

#### Synthesis and characterization of compound 1Me

Herein, we chose compound **1Me** as an analog of probe **1**. Compared to the latter, the former has an additional methyl group on the benzene ring. According to the synthesis method in the literature,^[1]^ the compound **1Me** was synthesized as the similar route shown in Scheme S1. And its structure is well characterized.

Characterization data: 0.8588 g, yield 85%; m.p.: 177.3°C–178.5°C: ^**1**^**H NMR** (600 MHz, CDCl_3_), *δ*, ppm: 2.42 (*s*, 3H), 2.46 (*s*, 3H), 6.65 (*s*, 1H), 7.35 (*d*, *J* = 8.4 Hz, 1H), 7.51 (*d*, *J* = 8.4 Hz, 1H), 8.63 (*s*, 1H); **ESI-HRMS**, *m*/*z*: Calcd for C_14_H_11_N_2_O [M + H]^+^, 223.0866, found: 223.0864.

#### Synthesis and characterization of 1Me-PyD

The compound **1Me-PyD** was obtained in the reaction shown as Scheme S3, which is basically similar with Scheme S2 (the synthetic route of compound **1-PyD**).

The compound **1Me** (0.222 g, 1 mmol) was dissolved in acetonitrile (5 mL), and pyrrolidine (82 *μ*L, 1 mmol) was added. In order to speed up the reaction, the reaction was refluxed for 30 min, the reaction solution was concentrated under reduced pressure. And the crude product was purified by silica gel column chromatography to give **1Me-PyD** as a yellowish solid.

Characterization data: m.p. 142.9°C–143.7°C; ^**1**^**H NMR** (600 MHz, CDCl_3_), *δ*, ppm: 1.91 (*t*, *J* = 6.6 Hz, 4H), 2.38 (*s*, 3H), 2.48 (*s*, 3H), 3.55 (*t*, *J* = 6.6 Hz, 4H), 6.95 (*s*, 1H), 7.12 (*d*, *J* = 8.4 Hz, 1H), 7.23 (*d*, *J* = 8.4 Hz, 1H), 7.64 (*s*, 1H); ^**13**^**C NMR** (150 MHz, CDCl_3_), *δ*, ppm: 21.0, 25.2, 25.5, 96.3, 103.4, 116.8, 123.7, 132.3, 133.4, 144.7, 150.7, 158.1, 158.2, 161.8; **ESI-HRMS**, *m*/*z*: Calcd for C_18_H_18_N_3_O [M-H]^-^, 292.1455, found: 292.1460.

Therefore, the structure of compound **1Me-PyD** is well characterized, and the characterization spectra (^1^H, ^13^C NMR and ESI-HRMS) can be seen in the following part, which are also included in this file.

### Quantification and statistical analysis

This study does not involve quantification and statistics.

### Additional resources

There are no additional resources need to be declared in this manuscript, additional requests for this can be made by contacting the lead contact.

## Supplemental information

Document S1. Figures S1–S19, Tables S1, S2 and Schemes S1–S3
