# Supplementary material for: The first specific probe for pyrrolidine with multifunction by the interaction mechanism of atomic economic reaction
Source: iScience. 2024 May 18;27(7):110024. doi: 10.1016/j.isci.2024.110024 (PMC11228642; doi:10.1016/j.isci.2024.110024)
Supplement: Document S1. Figures S1–S19, Tables S1, S2 and Schemes S1–S3 [file mmc1.pdf]

iScience, Volume *27*

Supplemental information

The first specific probe for pyrrolidine with multifunction by the interaction mechanism of atomic economic reaction

Xi-Ying Cao, Yan Huang, Si-Hong Chen, Shi-Wei Yu, Zu-Jia Chen, Zhong-Hao Li, Yu Zeng, Nan Chen, Liang Cao, and Zhao-Yang Wang

Supplemental information

The First Specific Probe for Pyrrolidine with Multifunction by the Interaction Mechanism of Atomic Economic Reaction

Xi-Ying Cao,†[1] Yan Huang,†[2] Si-Hong Chen,[1] Shi-Wei Yu,[1] Zu-Jia Chen,[1] Zhong-Hao Li,[1] Yu Zeng,[1] Nan Chen,[2,*] Liang Cao,[1,3,*] and Zhao-Yang Wang[1,*]

[1] *School of Chemistry, South China Normal University; Key Laboratory of Theoretical Chemistry of Environment, Ministry of Education; Guangzhou Key Laboratory of Analytical Chemistry for Biomedicine; GDMPA Key Laboratory for Process Control and Quality Evaluation of Chiral Pharmaceuticals, Guangzhou 510006, P. R. China*

[2] *The Education Ministry Key Lab of Resource Chemistry, Joint International Research Laboratory of Resource Chemistry of Ministry of Education, Shanghai Key Laboratory of Rare Earth Functional Materials, College of Chemistry and Materials Science, Shanghai Normal University, Shanghai 200234, P. R. China*

[3] *Shenzhen Key Laboratory of Cross-Coupling Reactions, Guangming Advanced Research Institute, Southern University of Science and Technology, Shenzhen, 518055, P. R. China*

‡These authors contributed equally to this work.

*Corresponding authors: wangzy@scnu.edu.cn; nchen@shnu.edu.cn; caol@sustech.edu.cn

Contents

Scheme S1. Synthetic pathway of probe **1**······3
Scheme S2. Synthetic pathway of compound **1-PyD**······4
Table S1. Crystal data and structure refinement for compound **1-PyD**······5
Fig. S1. Crystal structure of compound **1-PyD**······6
Fig. S2. Response time of fluorescence intensity······7
Fig. S3. Anti-interference experiment of probe **1**······8
Table S2. The comparison of our probe **1** with other sensors······9
Scheme S3. Synthetic route of compound **1Me-PyD**······11
Fig. S4. The molecular structures and packing mode in the **1-PyD** crystal······12
Fig. S5. Fluorescence changes of **1-PyD** in different water content systems······13
Fig. S6. Survival rates of zebrafishes treated with different concentrations of probe **1**······14
Fig. S7. ^{1}H NMR spectrum of compound **III**······15
Fig. S8. HRMS spectrum of compound **III**······16
Fig. S9. ^{1}H NMR spectrum of probe **1**······17
Fig. S10. HRMS spectrum of probe **1**······18
Fig. S11. ^{1}H NMR spectrum of compound **1-PyD**······19
Fig. S12. ^{13}C NMR spectrum of compound **1-PyD**······20
Fig. S13. HRMS spectrum of compound **1-PyD**······21
Fig. S14. HRMS spectrum of the reaction solution between probe 1 and PyD······22
Fig. S15. ^{1}H NMR spectrum of compound **1Me**······23
Fig. S16. HRMS spectrum of compound **1Me**······24
Fig. S17. ^{1}H NMR spectrum of compound **1Me-PyD**······25
Fig. S18. ^{13}C NMR spectrum of compound **1Me-PyD**······26
Fig. S19. HRMS spectrum of compound **1Me-PyD**······27
References······28

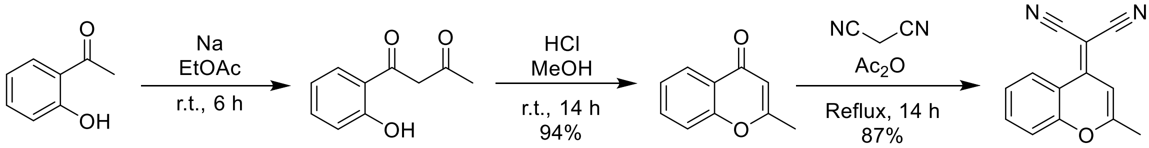

I II III 1

Scheme S1. Synthetic pathway of probe **1**. Related to **Scheme 1**

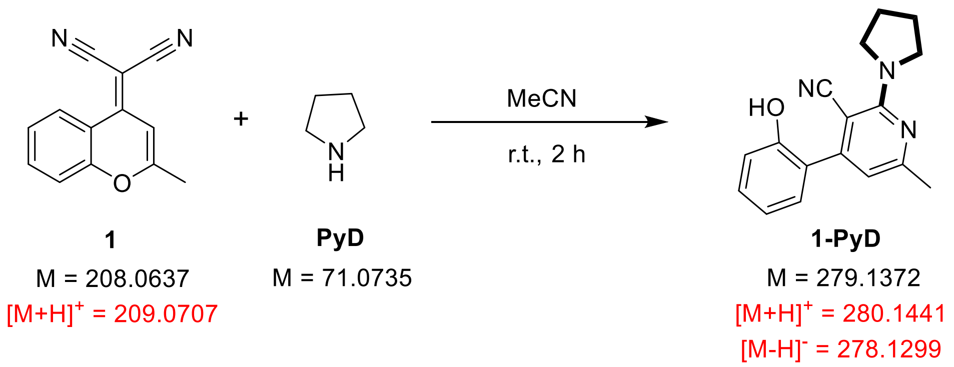

Scheme S2. Synthetic pathway of compound **1-PyD**. Related to **Scheme 1**

Table S1. Crystal data and structure refinement for compound **1-PyD**. Related to **Scheme 1**

Compound	**1-PyD**
Empirical formula	$C_{17}H_{17}N_3O$
Formula weight	279.33
Temperature/K	297
Crystal system	Monoclinic
Space group	P2/c
Unit cell dimensions (Å, °)	a = 9.6000(12), b = 7.8573(9), c = 20.413(2)
	α = 90, β = 102.853(12), γ = 90
Volume/Å^3	1501.2(3)
Z	4
Density (calculated) (Mg/cm^3)	1.236
Absorption coefficient (mm^{-1})	0.079
F(000)	592.00
Theta range for data collection/°	7.35 to 49.996
Index ranges	-11<=h<=11, -7<=k<=9, -24<=l<=24
Reflections collected	6387
Independent reflections	2644 [R_{int} = 0.0260, R_{sigma} = 0.0351]
Absorption correction	Semi-empirical from equivalents
Max. and min. transmission	1.000 and 0.519
Data / restraints / parameters	2644/0/192
Goodness-of-fit on F^2	1.028
Final R indexes [I>=2σ (I)]	R_1 = 0.0548, wR_2 = 0.1280
Final R indexes [all data]	R_1 = 0.0745, wR_2 = 0.1405
Extinction coefficient	n/a
Largest diff. peak / hole (e Å^{-3})	0.15 / -0.18

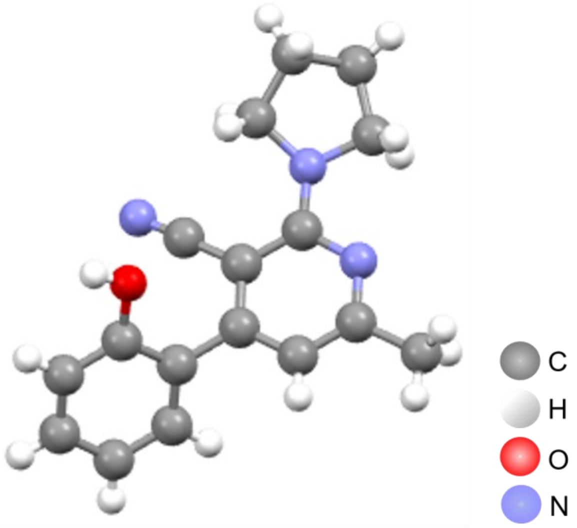

Fig. S1. Crystal structure of compound **1-PyD**. Related to **Scheme 1**

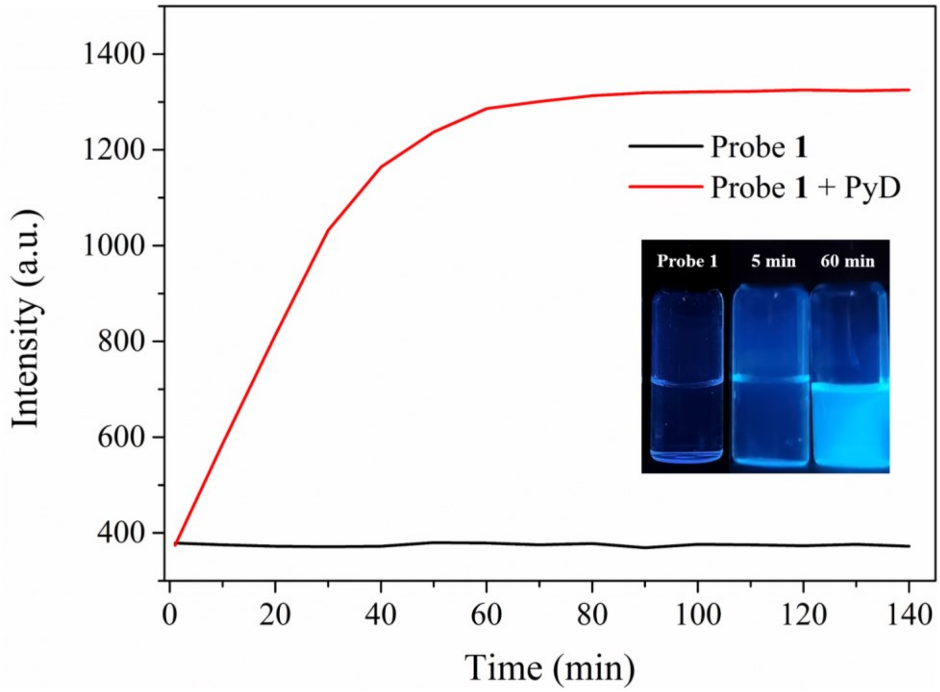

Fig. S2. Response time of fluorescence intensity (Inset: fluorescence color change of 5 min and 60 min, respectively). Related to **Figure 4.**

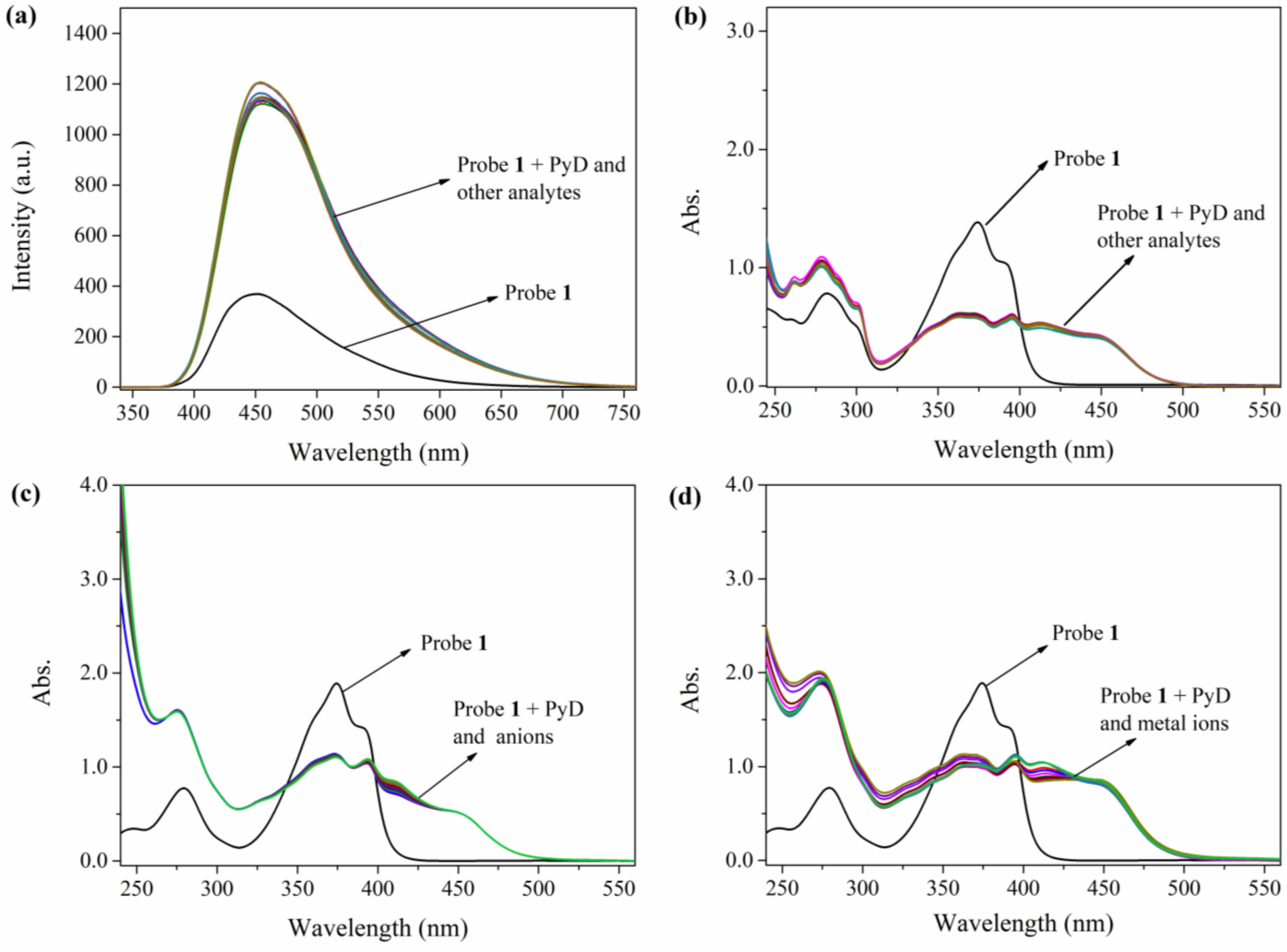

Fig. S3. Fluorescence emission spectra (a) (λ_{ex} = 275 nm) and UV absorption spectra (b) of probe 1 solution (10^{-4} M in MeCN) in the presence of 10 equiv. PyD and other organic analytes (10 equiv.), anions, including F^-, Cl^-, Br^-, I^-, NO_3^-, CN^-, HSO_4^-, $H_2PO_4^-$, AcO^-, ClO_4^-, BF_4^- (c), and metal ions, including Na^+, K^+, Ag^+, Mg^{2+}, Ca^{2+}, Ba^{2+}, Mn^{2+}, Cu^{2+}, Cd^{2+}, Hg^{2+}, Fe^{2+}, Pb^{2+}, Zn^{2+}, Ni^{2+}, Co^{2+}, Al^{3+}, Cr^{3+}, Fe^{3+} (d) (10 equiv.). Related to **Figure 3.**

Table S2. The comparison of probe **1** with reported probes for organic nitrogen-containing compounds. Related to **Figure 4.**

No.	Analyte	Response time	LOD	Signal type	Ref.
1	Lysine (Lys)<br>Arginine (Arg)	240 min<br>200 min	1.10×10^{-6} M<br>1.39×10^{-6} M	turn-on	1
2	Hydrazine	Not reported	1.22×10^{-5} M	ratio probe	2
3	Hydrazine	2 min	2.62×10^{-7} M	turn-on	3
4	Hydrazine	10 s	4.85×10^{-8} M	turn-on	4
5	Hydrazine	8 min<br>16 min	8.06×10^{-8} M<br>9.92×10^{-8} M	ratio probe<br>ratio probe	5
6	Hydrazine	Not reported	2.60×10^{-6} M	turn-on	6
7	Hydrazine	Real-time	1.68×10^{-7} M	ratio probe	7
8	Hydrazine	50 min	5.47×10^{-6} M	turn-on	8
9	Hydrazine	40 min	1.10×10^{-10} M	ratio probe	9
10	L-tryptophan	45 min	3.16×10^{-6} M	turn-on	10
11	Spermine<br>Spermidine	5 min<br>10 min	2.00×10^{-7} M<br>2.10×10^{-6} M	turn-off	11
12	L-glutamic acid<br>L-aspartic acid	Not reported	1.50×10^{-5} M<br>2.46×10^{-5} M	turn-off	12
13	$MeNH_2$<br>Lysine	5 min<br>5 min	6.90×10^{-9} M<br>6.20×10^{-8} M	ratio probe	13
14	Hydrazine	15 min	6.64×10^{-8} M	ratio probe	14
15	Hydrazine	40 s	4.80×10^{-8} M	ratio probe	15
16	Hydrazine	20 min	3.00×10^{-7} M	turn-on	16
17	Hydrazine	2 min	5.08×10^{-8} M	ratio probe	17
18	Hydrazine	7 min	2.71×10^{-7} M	turn-on	18
19	Hydrazine	Real-time	2.00×10^{-7} M<br>4.00×10^{-7} M<br>5.40×10^{-5} M	ratio probe	19

20	TEA<br>Ethylenediamine<br>Aniline<br>Hydrazine<br>PyD	Not reported	9.00×10^{-7} M<br>9.90×10^{-7} M<br>4.60×10^{-7} M<br>6.30×10^{-7} M<br>7.50×10^{-7} M	turn-off<br>ratio probe<br>turn-off<br>ratio probe<br>turn-off	20
This work	**PyD**	**< 5 min**	**1.12×10^{-6} M**	**turn-on**	

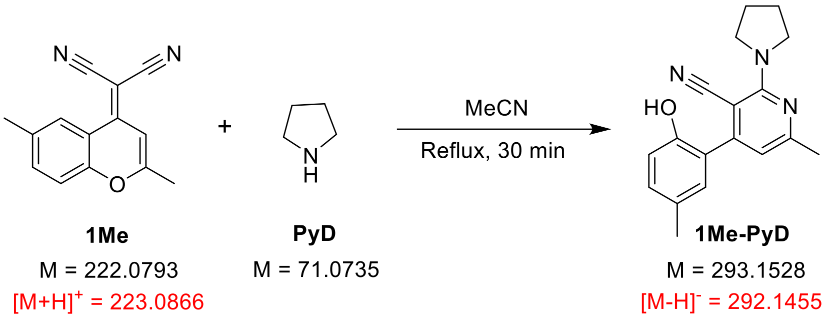

1Me M = 222.0793 [M+H]$^{+}$ = 223.0866

PyD M = 71.0735

1Me-PyD M = 293.1528 [M-H]$^{-}$ = 292.1455

Scheme S3. Synthetic route of compound **1Me-PyD**. Related to **Scheme 2.**

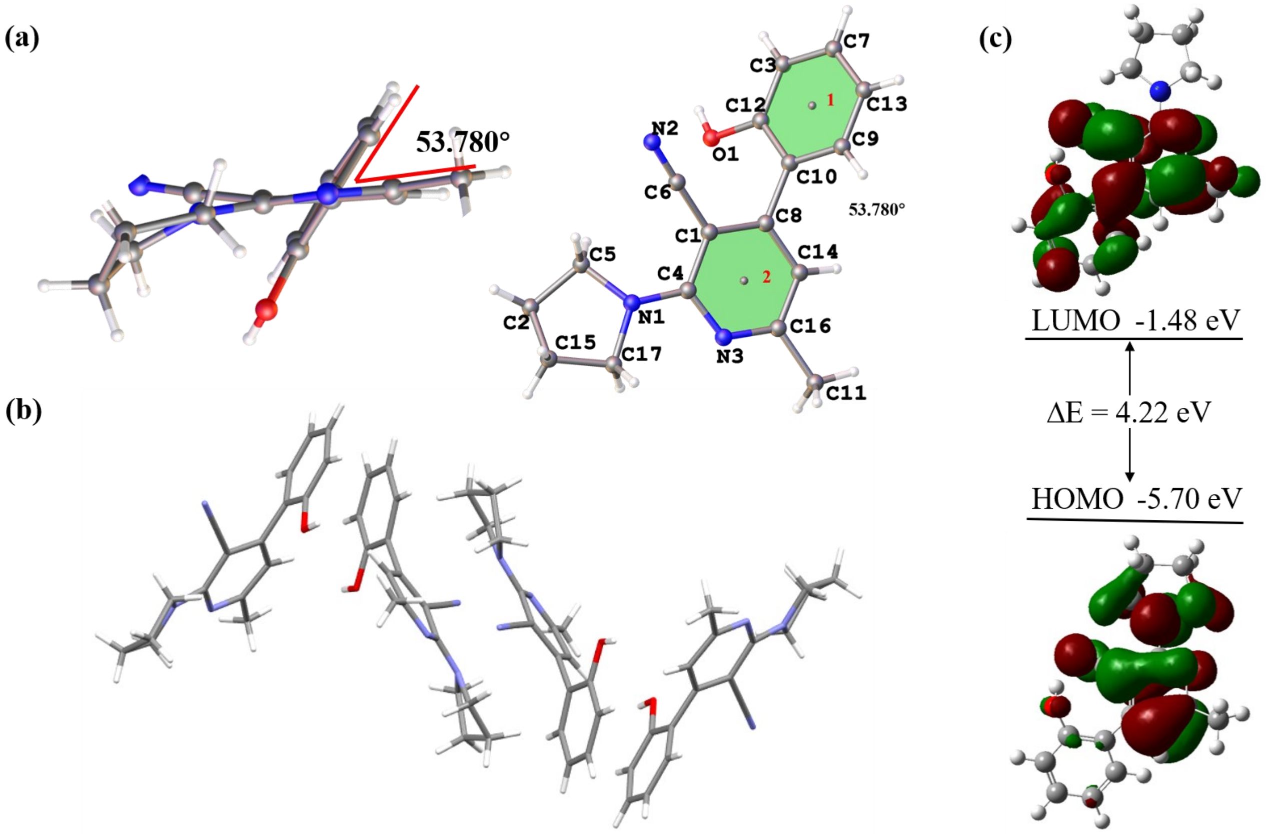

Fig. S4. The molecular structures and packing mode in the **1-PyD** crystal. (a) The dihedral angles between benzene ring (1) and pyridine ring (2); (b) The stacking mode of **1-PyD** aggregates; (c) HOMO and LUMO energy levels based on DFT calculations at the B3LYP/6-311 G(d, p). Related to **Scheme 2.**

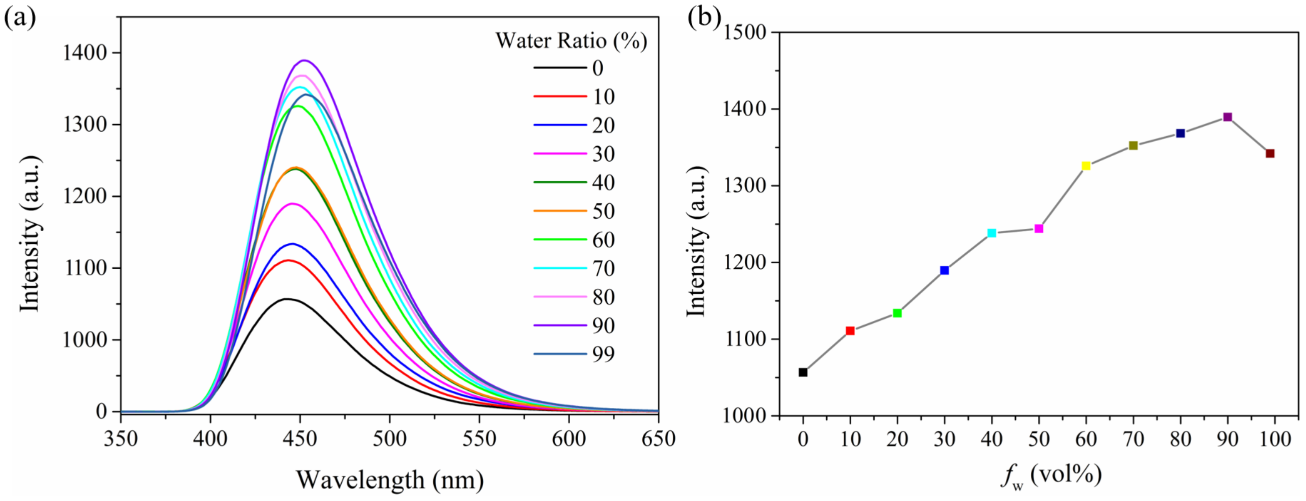

Fig. S5. The fluorescence spectra of compound **1-PyD** (10^{-5} M) in MeCN/H_2O mixture with increasing water fractions (*fw*) 0% to 99% (a) and line chart (b). Related to **Scheme 2.**

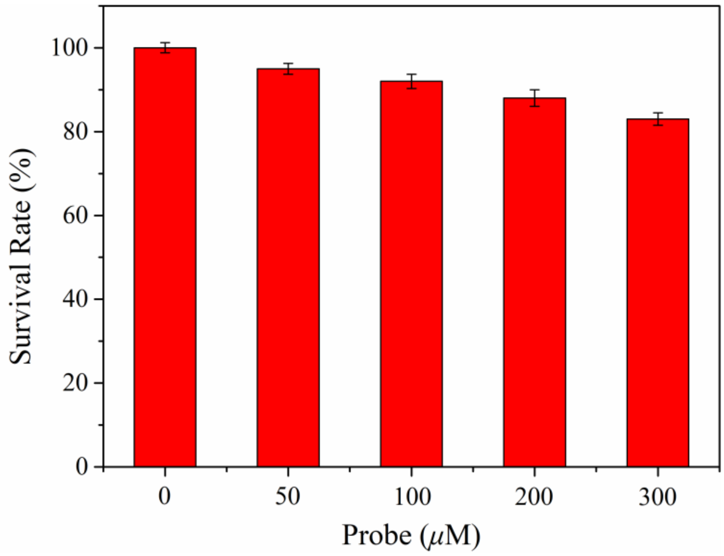

Fig. S6. Survival rates of zebrafishes treated with different concentrations of probe **1** (0, 50, 100, 200, 300 μM). Related to **Figure 6.**

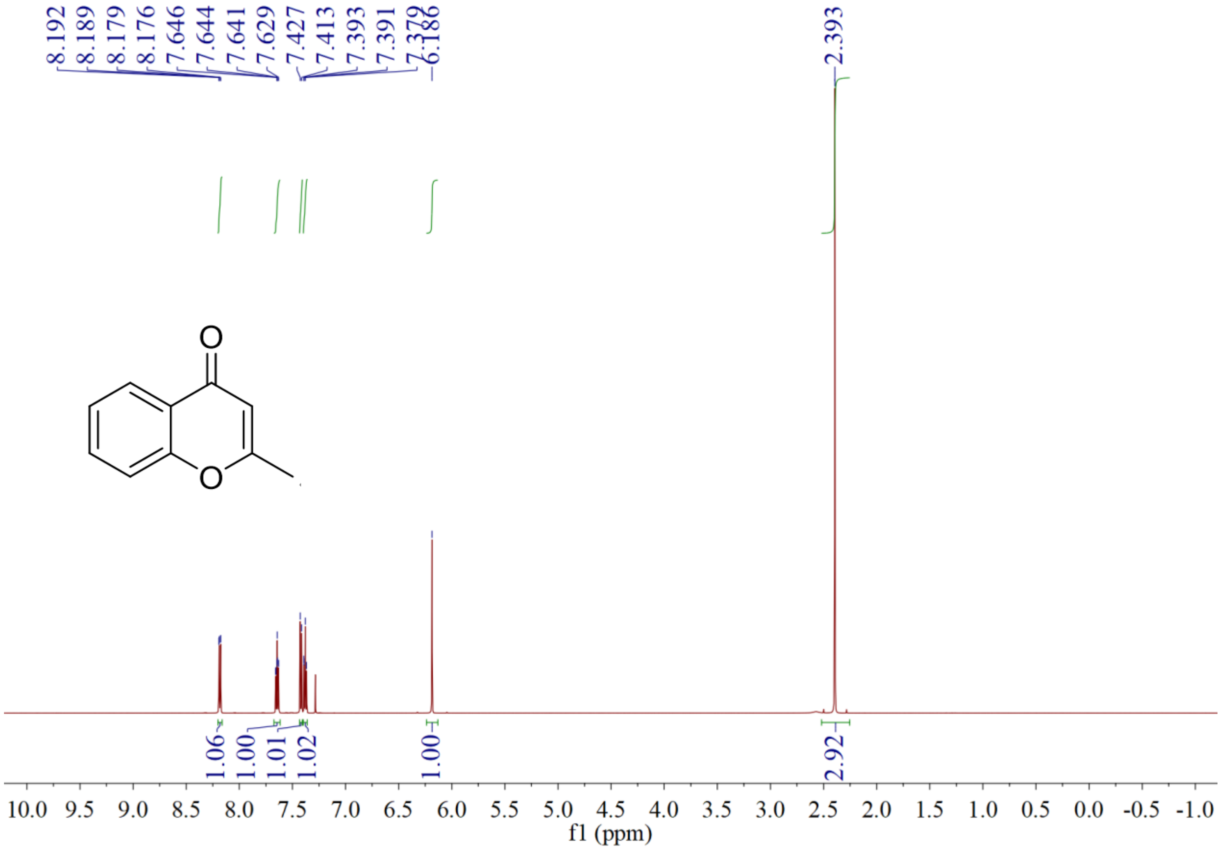

Fig. S7. ^{1}H NMR spectrum of compound **III.** Related to **Scheme 1.**

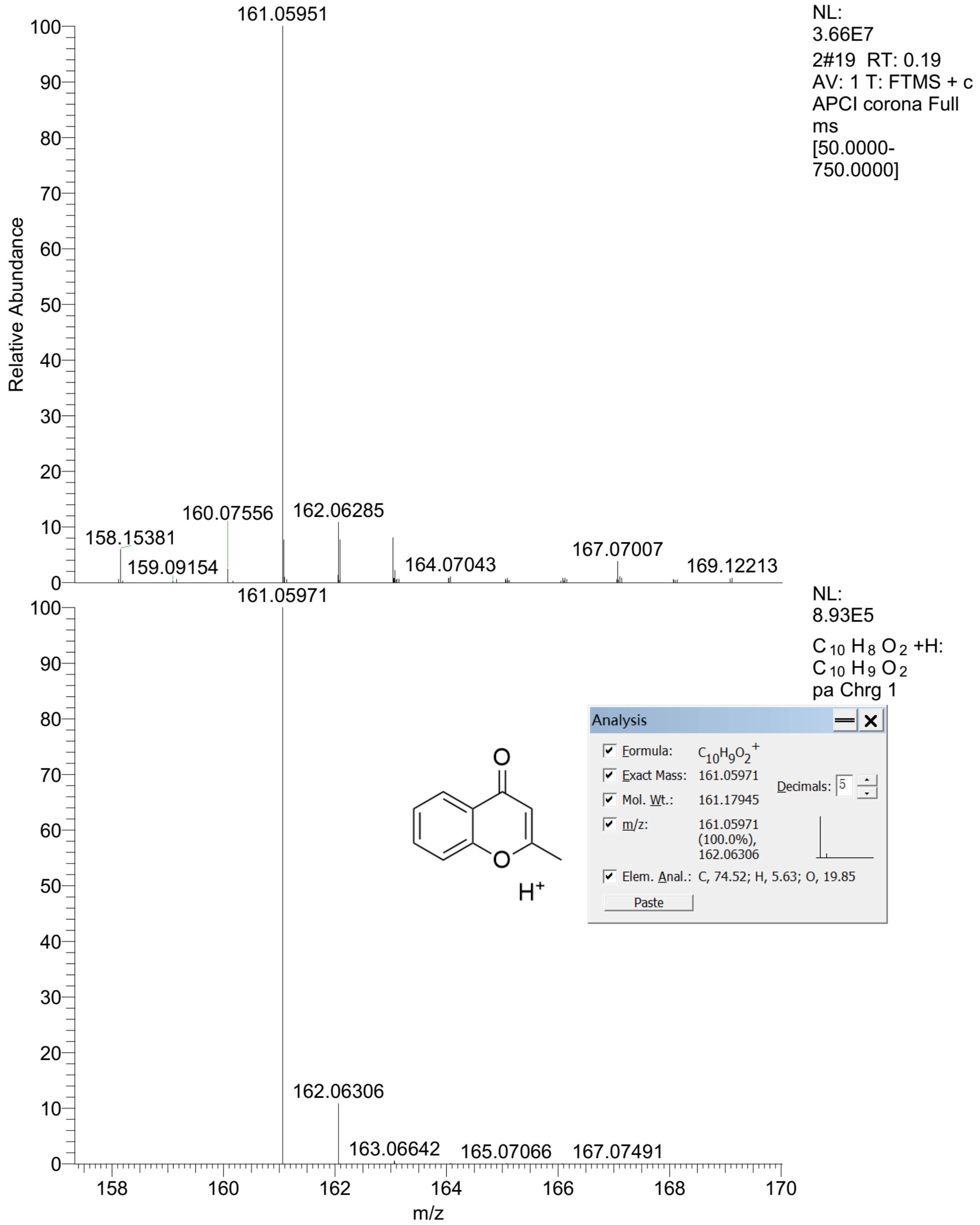

Fig. S8. HRMS spectrum of compound **III.** Related to **Scheme 1.**

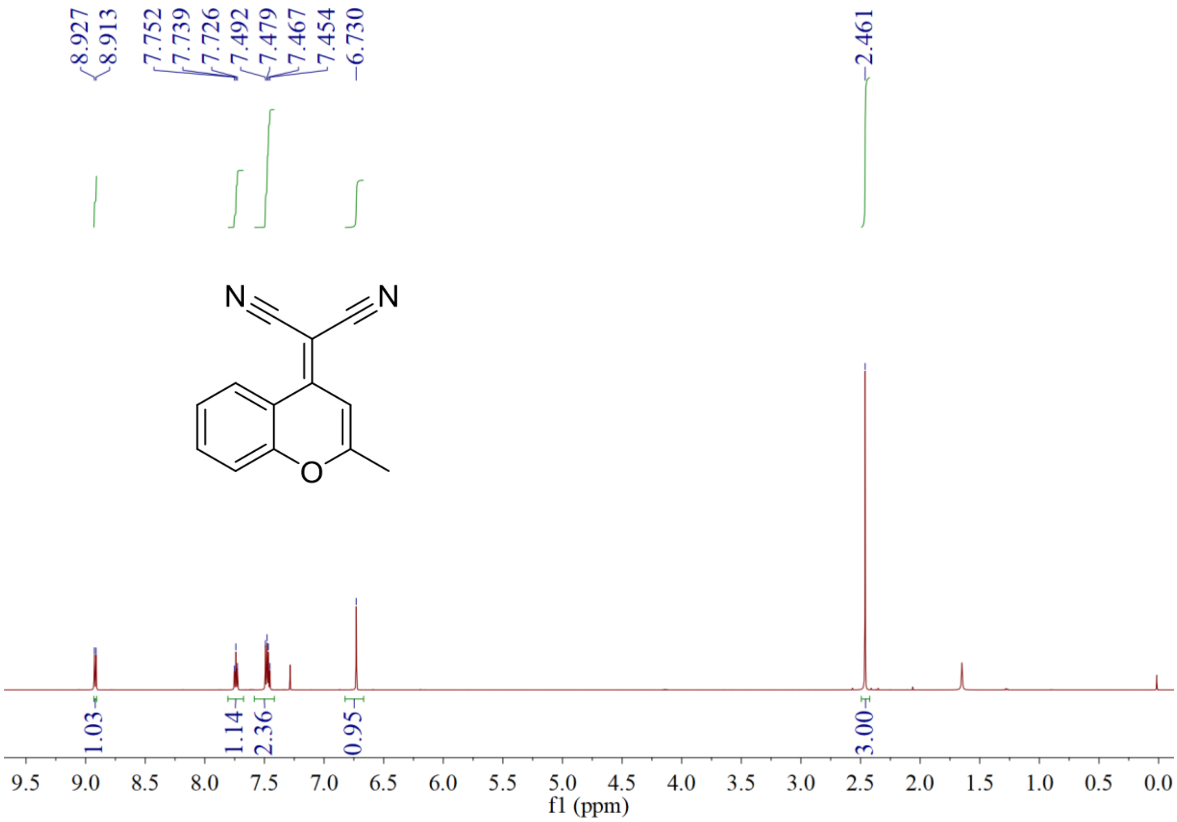

Fig. S9. ^{1}H NMR spectrum of probe **1.** Related to **Scheme 1.**

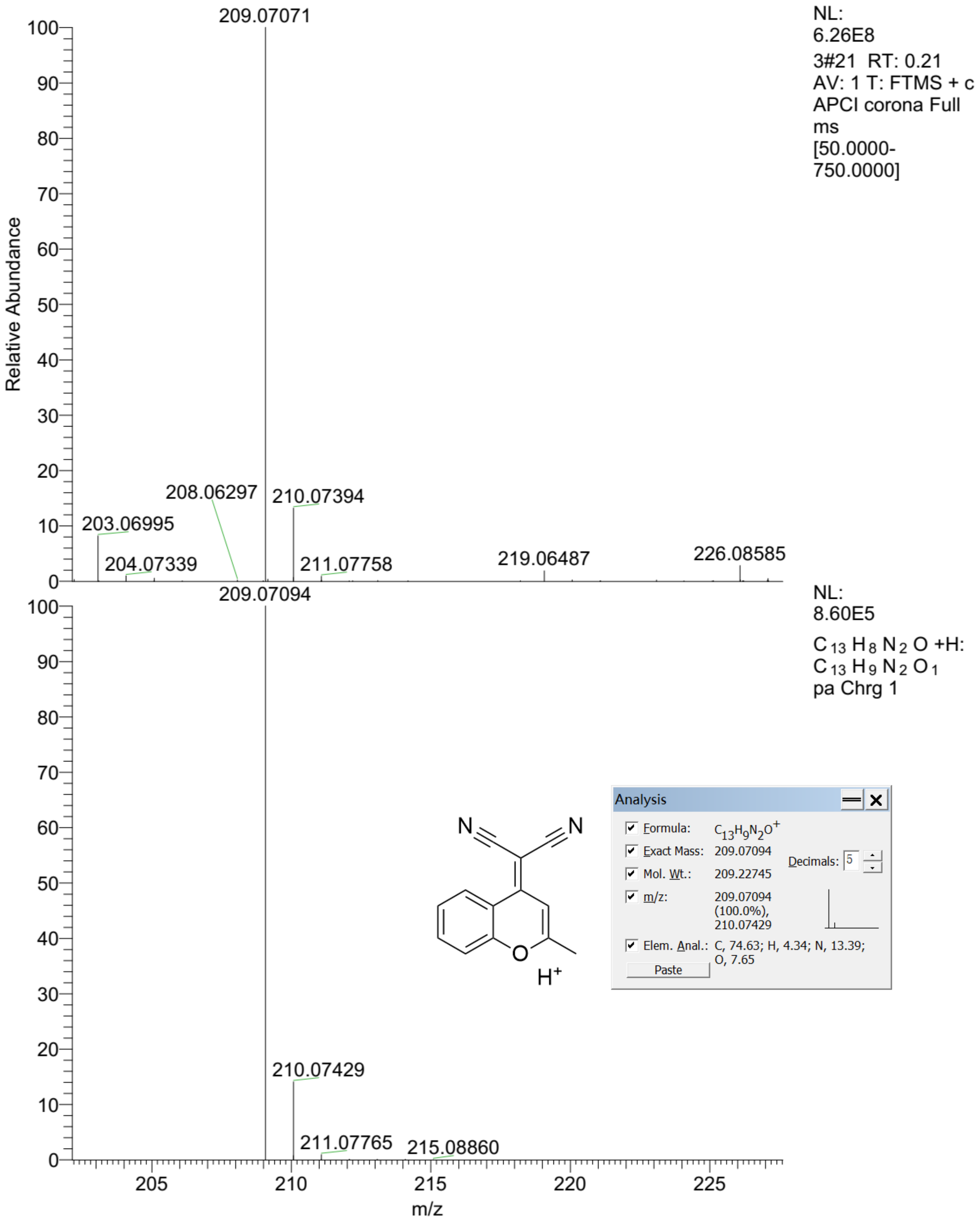

Fig. S10. HRMS spectrum of probe **1**. Related to **Scheme 1.**

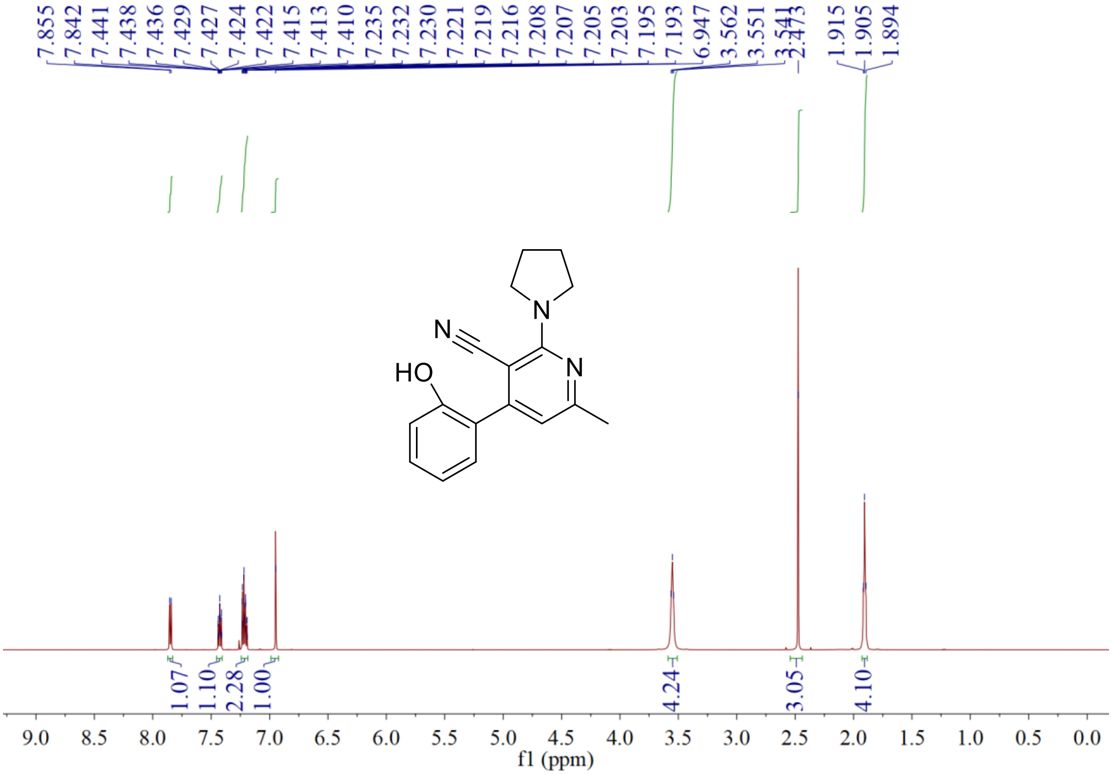

Fig. S11. ^{1}H NMR spectrum of compound **1-PyD.** Related to **Scheme 1.**

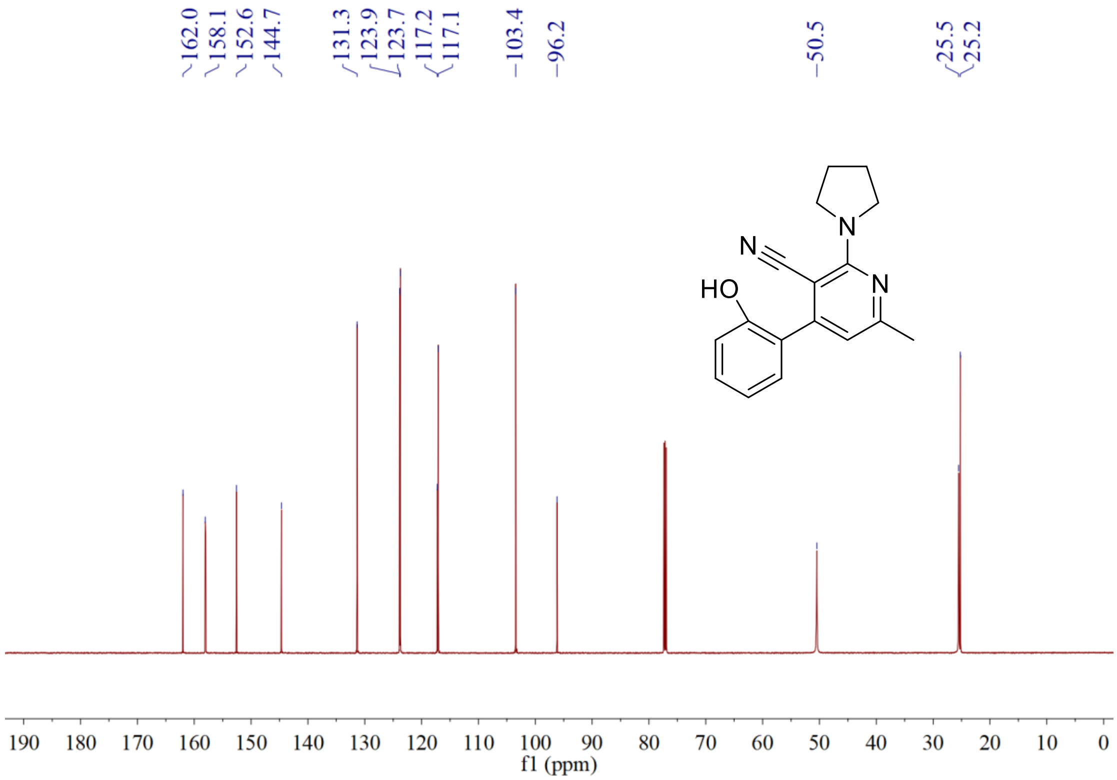

Fig. S12. ^{13}C NMR spectrum of compound **1-PyD.** Related to **Scheme 1.**

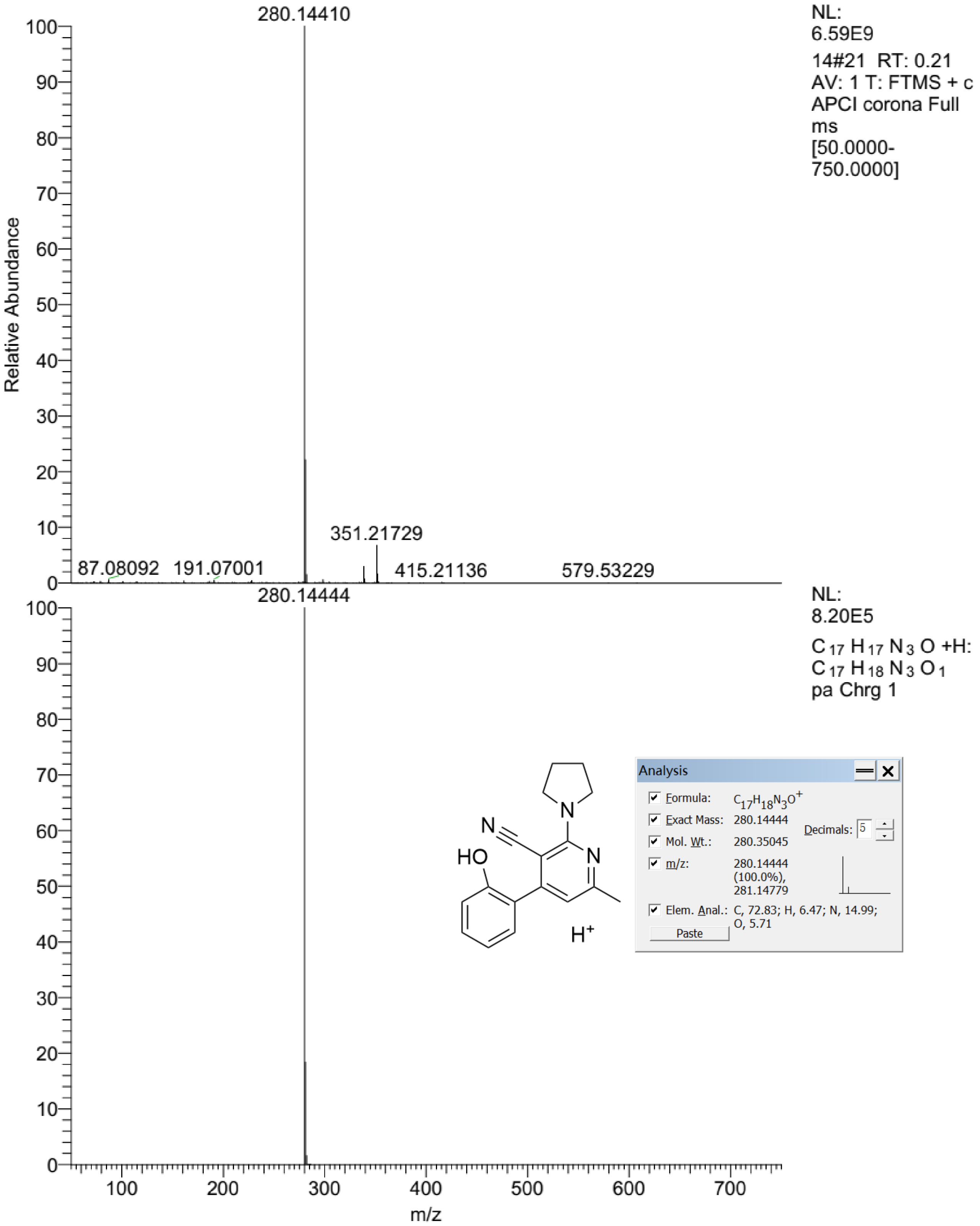

Fig. S13. HRMS spectrum of compound **1-PyD.** Related to **Scheme 1.**

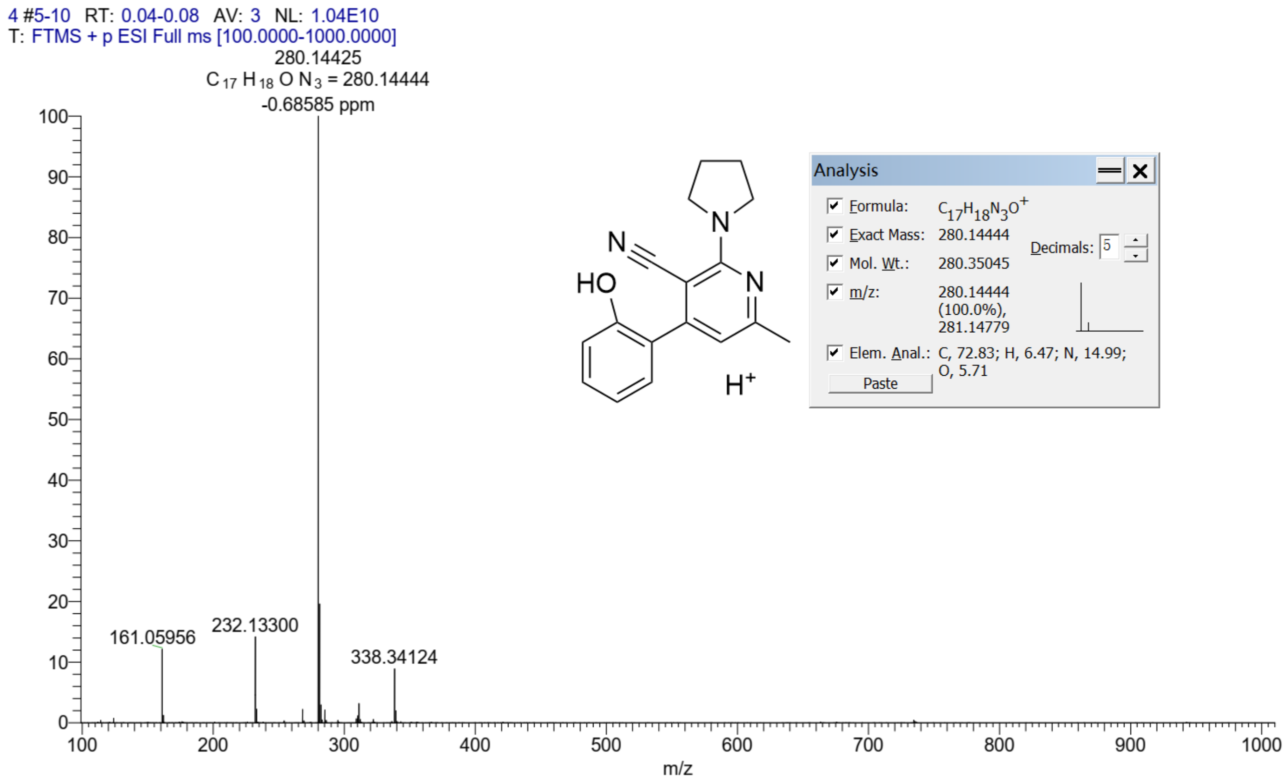

Fig. S14. HRMS spectrum of the reaction solution after 1 hour of interaction between probe **1** and **PyD**. Related to **Scheme 2.**

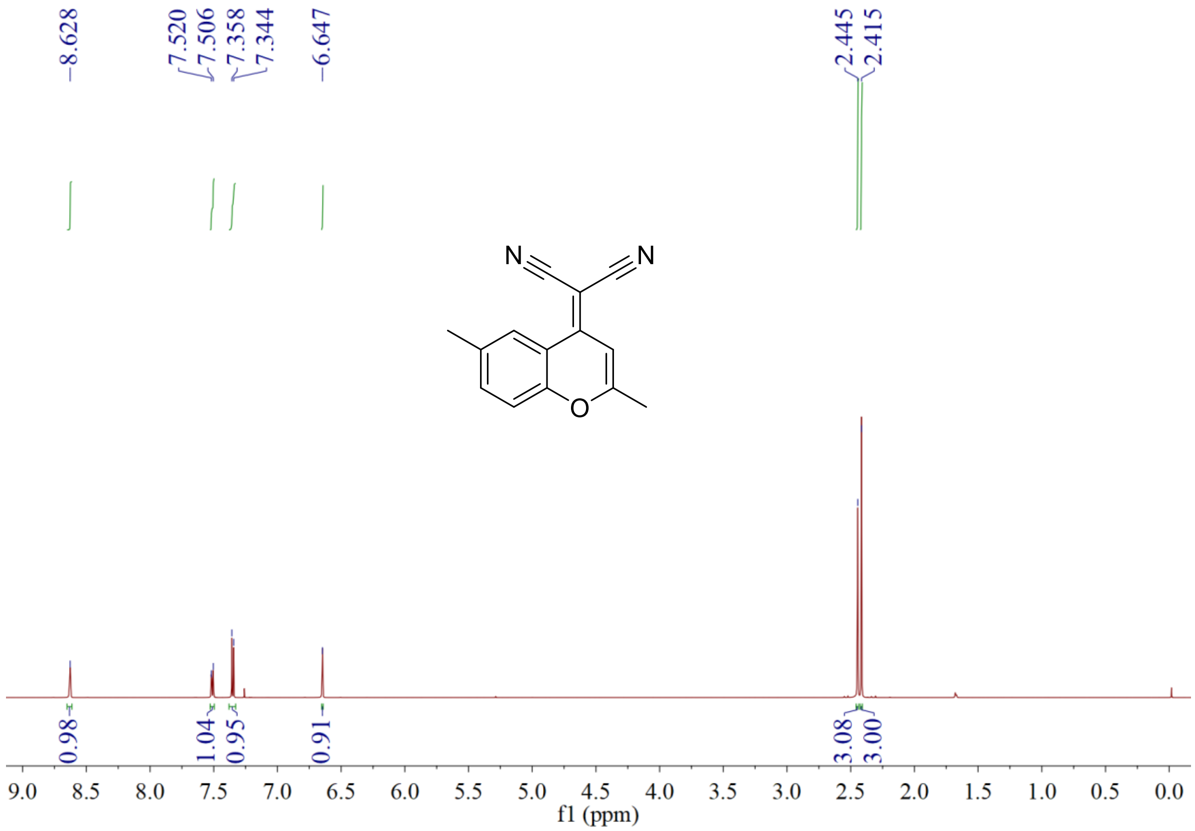

Fig. S15. ^{1}H NMR spectrum of compound **1Me.** Related to **Scheme 2.**

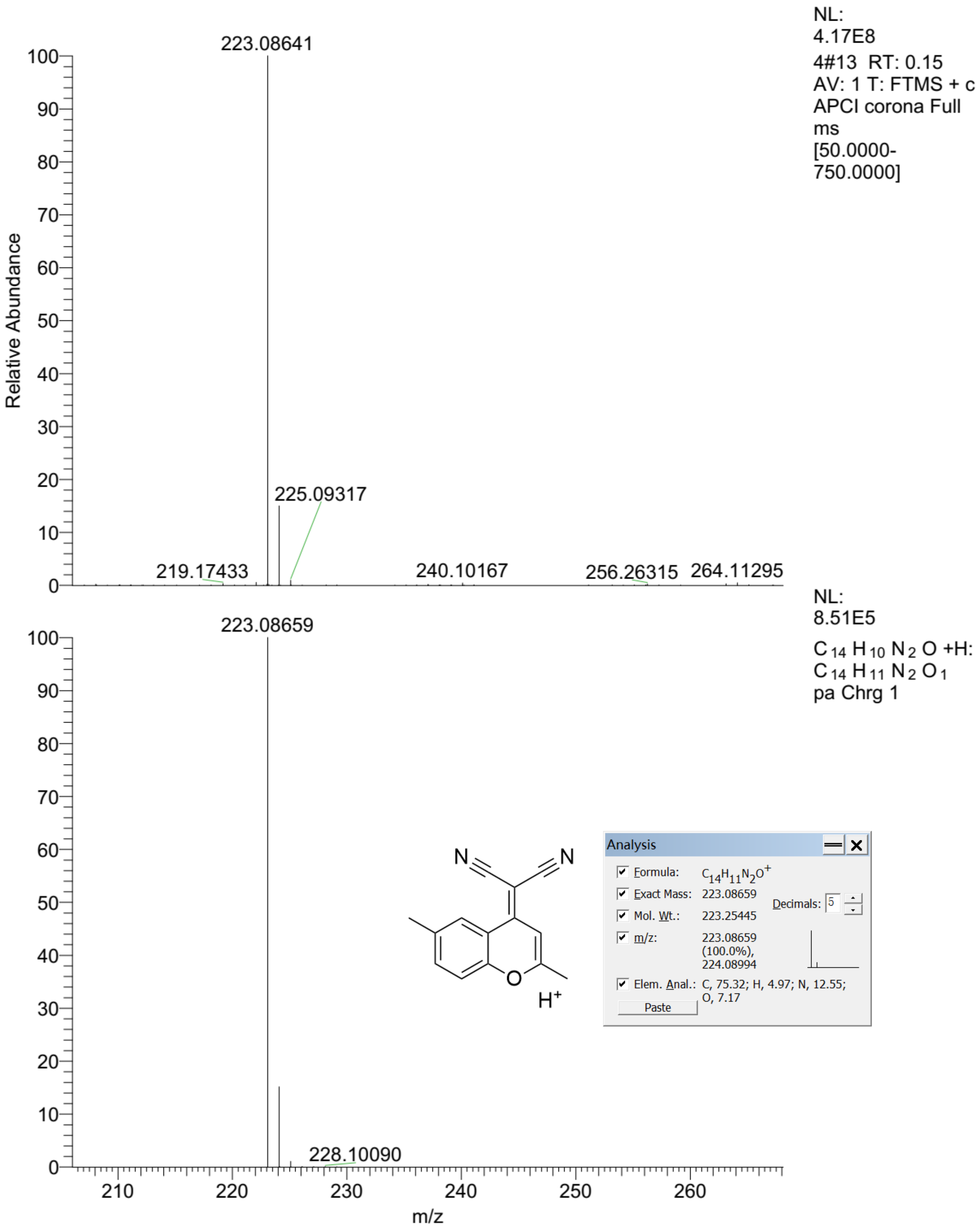

Fig. S16. HRMS spectrum of compound **1Me**. Related to **Scheme 2.**

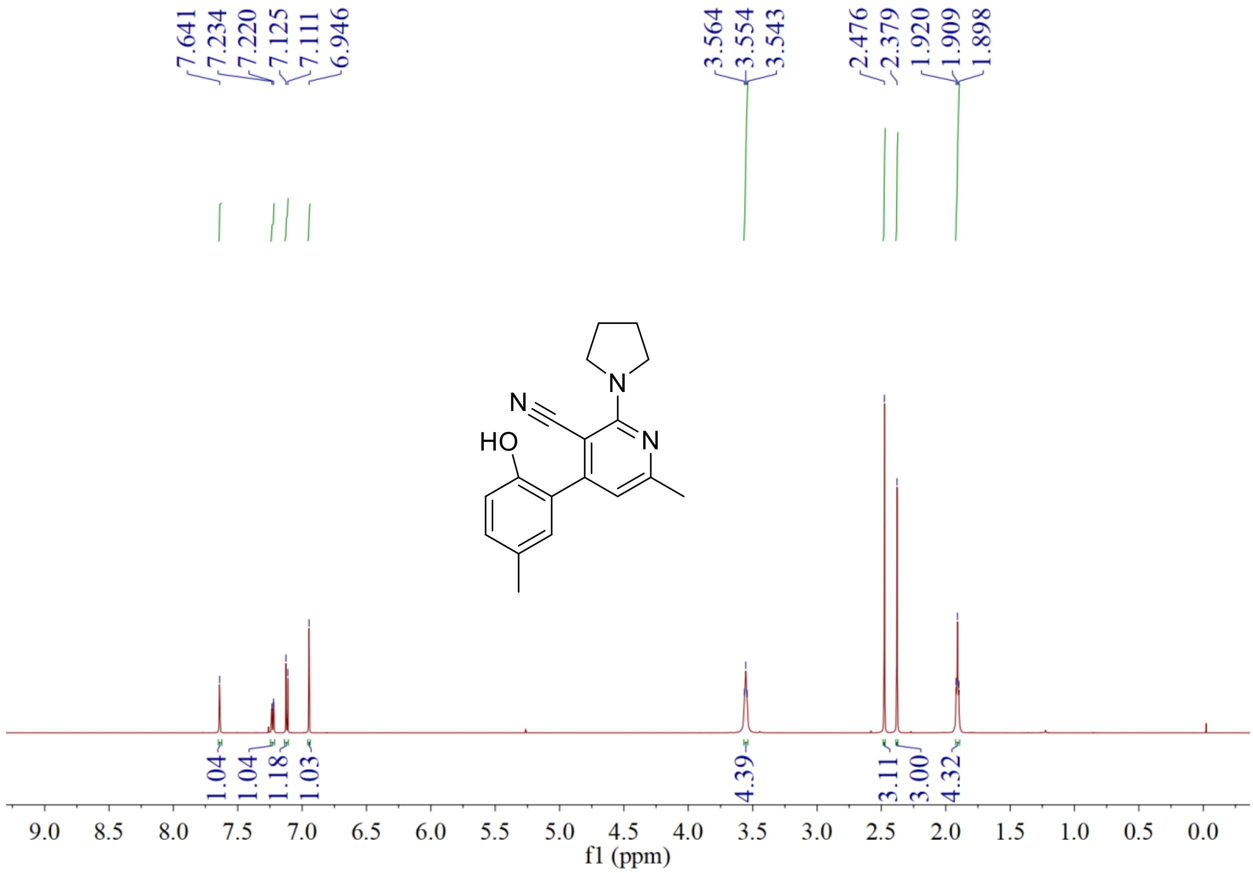

Fig. S17. ^{1}H NMR spectrum of compound **1Me-PyD**. Related to **Scheme 2.**

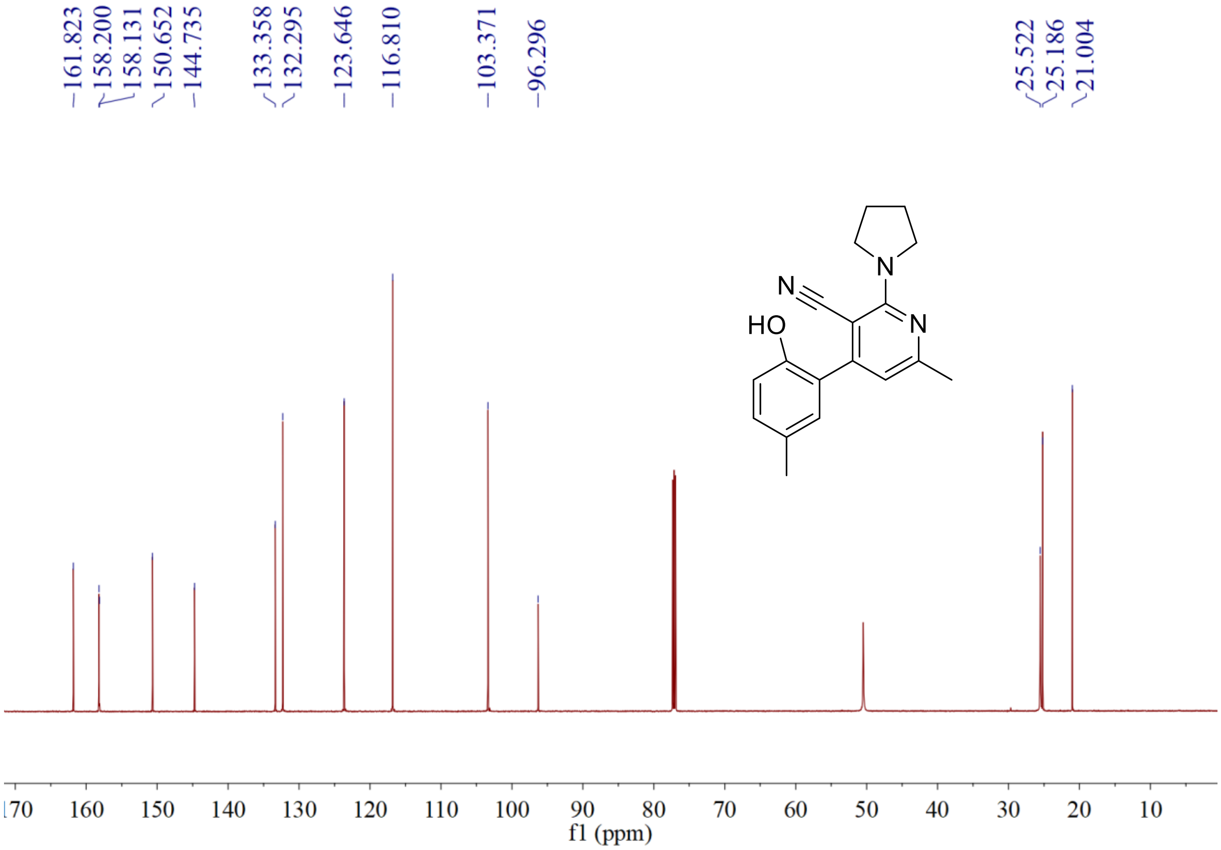

Fig. S18. ^{13}C NMR spectrum of compound **1Me-PyD**. Related to **Scheme 2.**

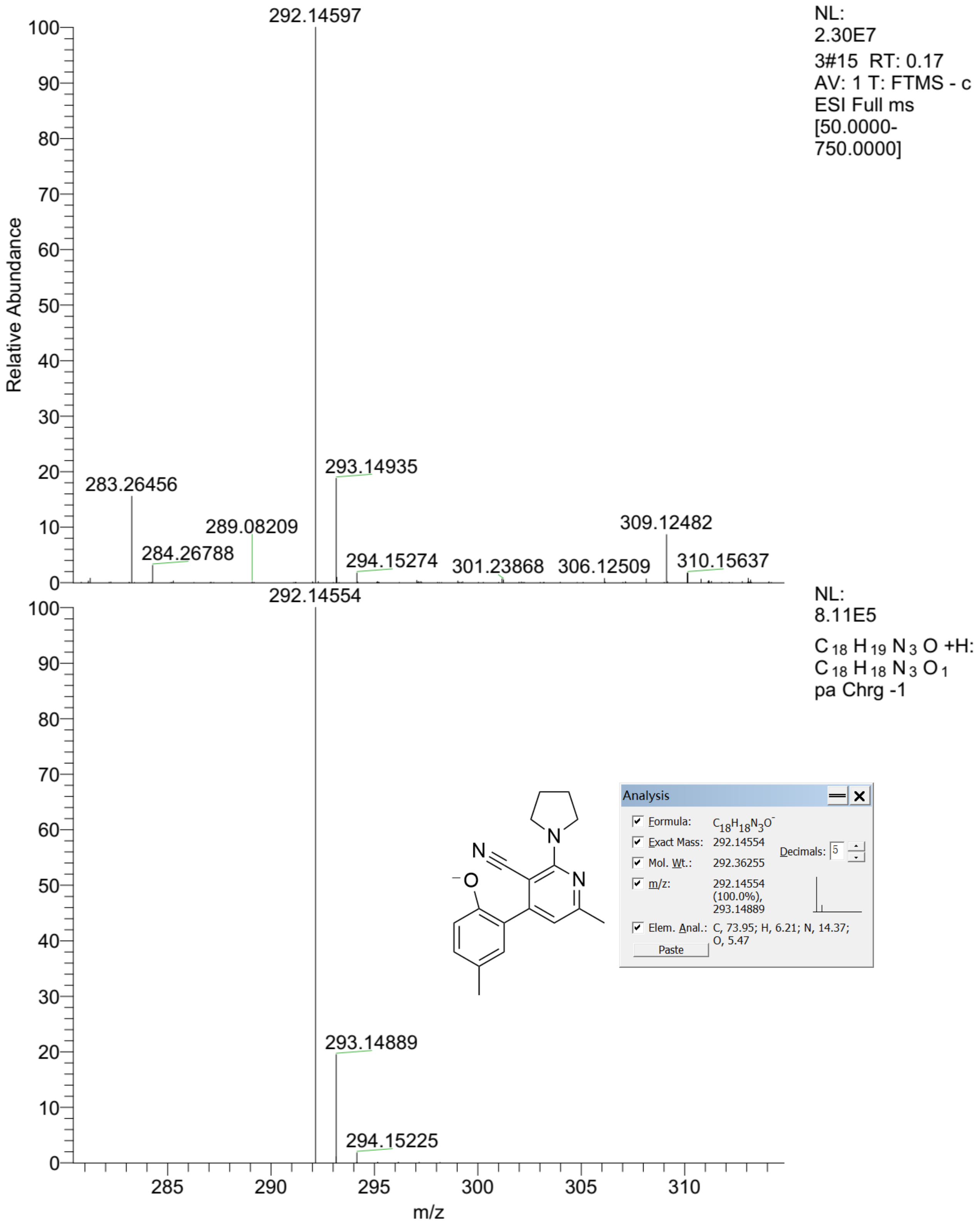

Fig. S19. HRMS spectrum of compound **1Me-PyD**. Related to **Scheme 2.**

References

1 Yang, L., Xie, Y. S., Chen, Q. X., Zhang, J., Li, L., and Sun, H. Y. (2021). Colorimetric and fluorescent dual-signal chemosensor for lysine and arginine and its application to detect amines in solid-phase peptide synthesis. ACS Appl. Bio. Mater. 4, 6558-6564.

2 Liu, T. H., Yang, L. J., Feng, W., Liu, K., Ran, Q., Wang, W. N., Liu, Q., Peng, H. N., Ding, L. P., and Fang, Y. (2020). Dual-mode photonic sensor array for detecting and discriminating hydrazine and aliphatic amines. ACS Appl. Mater. Interfaces 12, 11084-11093.

3 Pavankumar, B. B., Ranjan, P., Jha, P. C. and Sivaramakrishna, A. (2020). New simple molecular fluorescent probes for rapid and highly selective sensing of hydrazine by aggregate-induced emission. Analyst 145, 4615-4626.

4 Liu, S. S., Wu, W. N., Zhao, X. L., Fan, Y. C., Wang, Y., and Xu, Z. H. (2023). A dual-emission fluorescence probe for the detection of viscosity and hydrazine in environmental and biological samples. Anal. Chim. Acta 1245, 340867.

5 Erdemir, S., Malkondu, S. and Oguz, M. (2023). Fast, visual, and quantitative monitoring of N_2H_4 by two ratiometric fluorescent probes in environmental media and biological systems. Chem. Eng. J. 468, 143767.

6 Hou, J. T., Wang, B. Y., Wang, S., Wu, Y. Q., Liao, Y. X., and Ren, W. X. (2020). Detection of hydrazine via a highly selective fluorescent probe: A case study on the reactivity of cyano-substituted C=C bond. Dyes Pigm. 178, 108366.

7 Zhang, F., Li, D., Liang, X. Z., Guo, K. P., Li, B., Xu, H., Li, J., Zhang, Z., Wang, S. J., Fan, F., and Sun, Y. (2021). Forming luminescent oligomer nanoparticles via condensation polymerization: A strategy for real-time visualized detection of hydrazine in solution and gas phase. Dyes Pigm. 185, 108931.

8 Tang, L. J., Zhou, L., Liu, A. J., Yan, X. M., Zhong, K. L., Liu, X. Y., Gao, X., and Li, J. R. (2021). A new cascade reaction-based colorimetric and fluorescence "turn-on" dual-function probe for cyanide and hydrazine detection. Dyes Pigm. 186, 109034.

9 Zhang, T. G., Zhu, L. L., and Lin, W. Y. (2021). A near infrared ratiometric fluorescent probe with aggregation induced emission (AIE) characteristics for hydrazine detection in vitro and in vivo. Dyes Pigm. 188, 109177.

10 Liu, G. J., Wang, L. M., Zhu, F. W., Liu, Q., Feng, Y. H., Zhao, X. Y., Chen, M., and Chen, X. Q. (2022). Facile construction of a reusable multi-enzyme cascade bioreactor for effective fluorescence discrimination and quantitation of amino acid enantiomers. Chem. Eng. J. 428, 131975.

11 Abbasi-Moayed, S., Bigdeli, A., and Hormozi-Nezhad, M. R. (2022). Determination of spermine and spermidine in meat with a ratiometric fluorescence nanoprobe and a combinational logic gate. Food Chem. 384, 132459.

12 Han, T. T., Huang, Y., Gao, T. Y., Xia, C. Y., Sun, C., Xu, W. M., and Wang, D. Y. (2023). Fabrication of nitrogen-doped graphene quantum dots based fluorescent probe and its application for simultaneous, sensitive and selective detection of umami amino acids. Food Chem. 404, 134509.

13 Jeon, S., Kim, T. I., Jin, H., Lee, U., Bae, J., Bouffard, J., and Kim Y. (2020). Amine-reactive activated esters of meso-carboxy BODIPY: fluorogenic assays and labeling of amines, amino acids, and proteins. J. Am. Chem. Soc. 142, 9231-9239.

14 Wang, B. B., Yang, R. J., and Zhao, W. (2021), Construction of a mitochondria-targeted ratiometric fluorescent probe for monitoring hydrazine in soil samples and culture cells. J. Hazard. Mater. 406, 124589.

15 Wang, Z. L., Zhang, Y., Meng, Z. Y., Li, M. X., Zhang, C. L., Yang, L. J., Yang, Y. Q., Xu, X., and Wang, S. F. (2022). Development of a ratiometric fluorescent probe with large Stokes shift and emission wavelength shift for real-time tracking of hydrazine and its multiple applications in environmental analysis and biological imaging. J. Hazard. Mater. 422, 126891.

16 Zeng, C. H., Xu, Z. Y., Song, C., Qin, T. Y., Jia, T. H., Zhao, C., Wang, L., Liu, B., and Peng, X. J. (2023). Naphthalene-based fluorescent probe for on-site detection of hydrazine in the environment. J. Hazard. Mater. 445, 130415.

17 Erdemir, S., Oguz, M., and Malkondu, S. (2022). Real-time screening of hydrazine by a NIR fluorescent probe with low cytotoxicity in living cells and its multiple applications: optimization using box-behnken design. Sens. Actuators, B 364, 131893.

18 Cui, Y. L., Xu, C. G., Wu, T., Nie, Y. M., and Zhou, Y. M. (2022). Near-infrared cyanine-based fluorescent probe: Rapidly visualizing the in situ release of hydrazine in living cells and zebrafish.

Sens. Actuators, B 350, 130878.

19 Shang, Z. Y., Tian, S. Y., Wang, Y., Zhang, C., Meng, Q. T., Zhang, R., and Zhang, Z. Q. (2024). 1,8-naphthalimide-triphenylamine-based red-emitting fluorescence probes for the detection of hydrazine in real water samples and applications in bioimaging *in vivo*. Sens. Actuators, B 398, 134725.

20 Nawaz, H., Zhang, J. M., Tian, W. G., Jin, K. F., Jia, R. N., Yang, T. T., and Zhang, J. (2020). Cellulose-based fluorescent sensor for visual and versatile detection of amines and anions. J. Hazard. Mater. 387, 121719.
